# Supplementary material for: Isoforms of the TAL1 transcription factor have different roles in hematopoiesis and cell growth
Source: PLoS Biol. 2023 Jun 28;21(6):e3002175. doi: 10.1371/journal.pbio.3002175 (PMC10335695; doi:10.1371/journal.pbio.3002175)
Supplement: S1 Fig — (A and B) RNA was extracted from the indicated cell lines and analyzed by real-time PCR for total mRNA amount of TAL1 relative to CycloA and hTBP reference genes (S1 Data) (A) and for ΔEx3 relative to endogenous TAL1 total mRNA amount. PSI was calculated by ΔEx3 relative to endogenous TAL1 total mRNA (S1 Data) (B). (C) ChIP-seq tracks for H3K27ac and H3K4me3 at the TAL1 locus in the indicated cell-lines (genome build hg19). (PPTX) [file pbio.3002175.s001.pptx]

## Slide 1
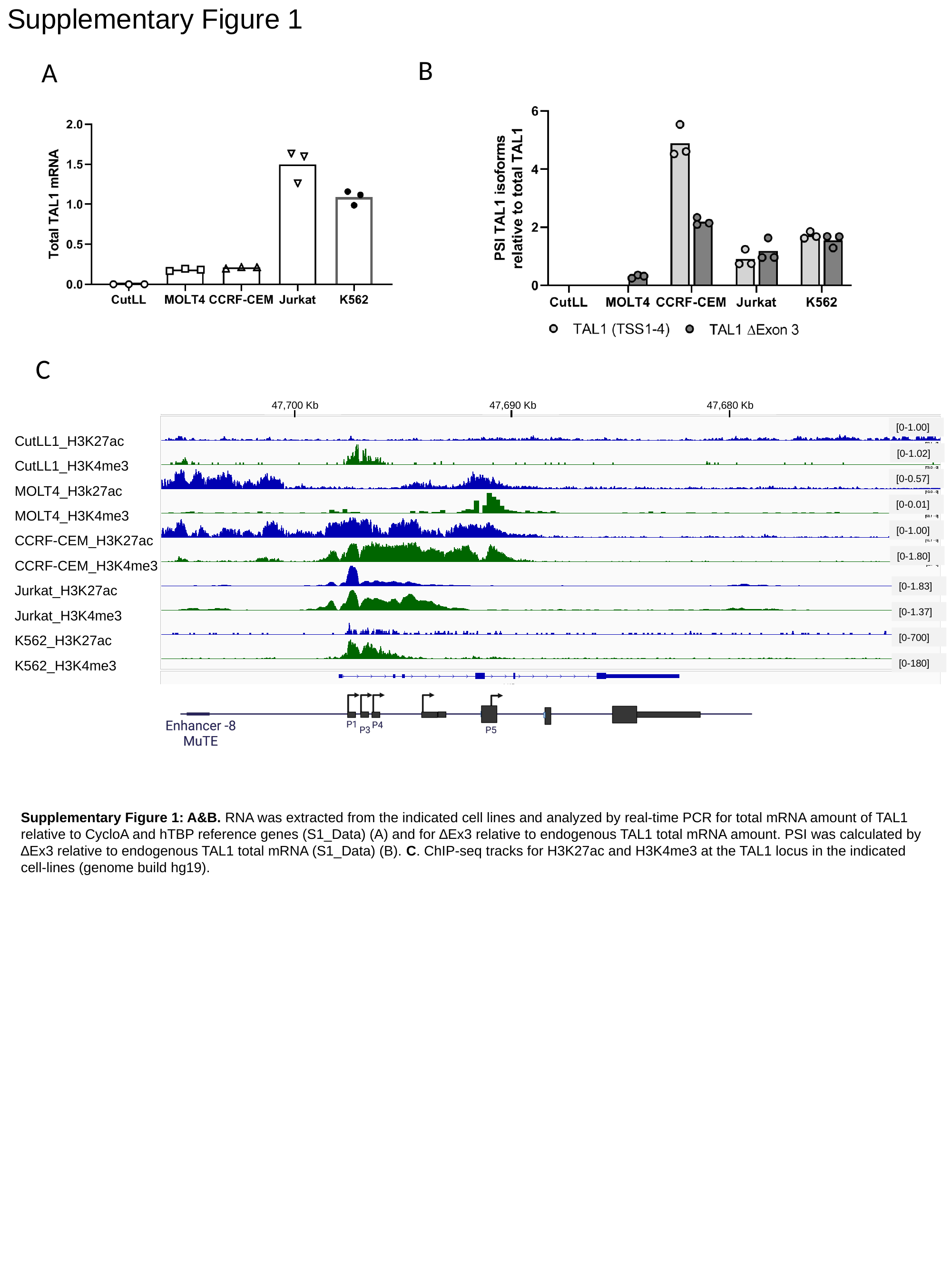

Supplementary Figure 1
B
A
C
47,700 Kb
47,690 Kb
47,680 Kb
[0-1.00]
CutLL1_H3K27ac
CutLL1_H3K4me3
MOLT4_H3k27ac
MOLT4_H3K4me3
CCRF-CEM_H3K27ac
CCRF-CEM_H3K4me3
Jurkat_H3K27ac
Jurkat_H3K4me3
K562_H3K27ac
K562_H3K4me3
[0-1.02]
[0-0.57]
[0-0.01]
[0-1.00]
[0-1.80]
[0-700]
[0-180]
[0-1.83]
[0-1.37]
Supplementary Figure 1: A&B. RNA was extracted from the indicated cell lines and analyzed by real-time PCR for total mRNA amount of TAL1 relative to CycloA and hTBP reference genes (S1_Data) (A) and for ∆Ex3 relative to endogenous TAL1 total mRNA amount. PSI was calculated by ∆Ex3 relative to endogenous TAL1 total mRNA (S1_Data) (B). C. ChIP-seq tracks for H3K27ac and H3K4me3 at the TAL1 locus in the indicated cell-lines (genome build hg19).
