# Supplementary material for: Isoforms of the TAL1 transcription factor have different roles in hematopoiesis and cell growth
Source: PLoS Biol. 2023 Jun 28;21(6):e3002175. doi: 10.1371/journal.pbio.3002175 (PMC10335695; doi:10.1371/journal.pbio.3002175)
Supplement: S2 Fig — (A) Sequencing of 5′ RACE PCR in Jurkat cells aligned to TAL1 locus. Peaks mark the sequence of the 5′ UTR. Red asterisk marks a peak that did not align to any known TSS. (B) Schematic representation of TAL1 mRNA isoforms. Rectangles: exons, black lines: introns; arrow: transcription initiation site. (C and D) Whole cell lysate was extracted from Jurkat NLO cells, expressing TAL1 exogenously, and enhancer mutated cells Jurkat Del-12, and subjected to western blot analysis using the indicated antibodies (S1 Raw Images) (C). RNA was extracted and real-time PCR was conducted TAL1 promoter relative to to CycloA and hTBP reference genes (S1 Data) (D). (E) Schematic representation of TAL1 genomic locus. Rectangles: exons, black lines: introns, arrow: transcription initiation site. (F-H) Whole cell lysate was extracted from HEK293T cells and CTCF mutated cells, HEK293T ΔCTCF, and subjected to western blot analysis using the indicated antibodies (S1 Raw Images) (F). RNA was extracted and analyzed by real-time PCR for each of TAL1’s promoters relative to CycloA and hTBP reference genes (S1 Data) (G). (H) HEK293T cells were transfected with either dCas9-p300 core (mut) or dCas9-p300 core (WT) with 4 gRNAs targeted to the TAL1 −60 enhancer for 30 h. Whole cell lysate was extracted and subjected to western blot analysis using the indicated antibodies (S1 Raw Images). (I and J) Jurkat cells were transfected with siKMT2B or a negative control siRNA (siGFP), and RNA was extracted 72 h posttransfection. Real-time PCR was performed to KMT2B total mRNA amount relative to CycloA and hTBP reference genes (S1 Data) (I) and total mRNA amount of TAL1 relative to CycloA and hTBP reference genes (S1 Data) (J). The mean was calculated from 3 independent biological experiments, each performed with 3 technical replicates. (K-M) Jurkat cells were treated with 6 μM CPT for 6 h and analyzed by real-time PCR for total mRNA amount of CycloA relative to 18S reference gene transcribed by Polymerase I [file pbio.3002175.s002.pptx]

## Slide 1
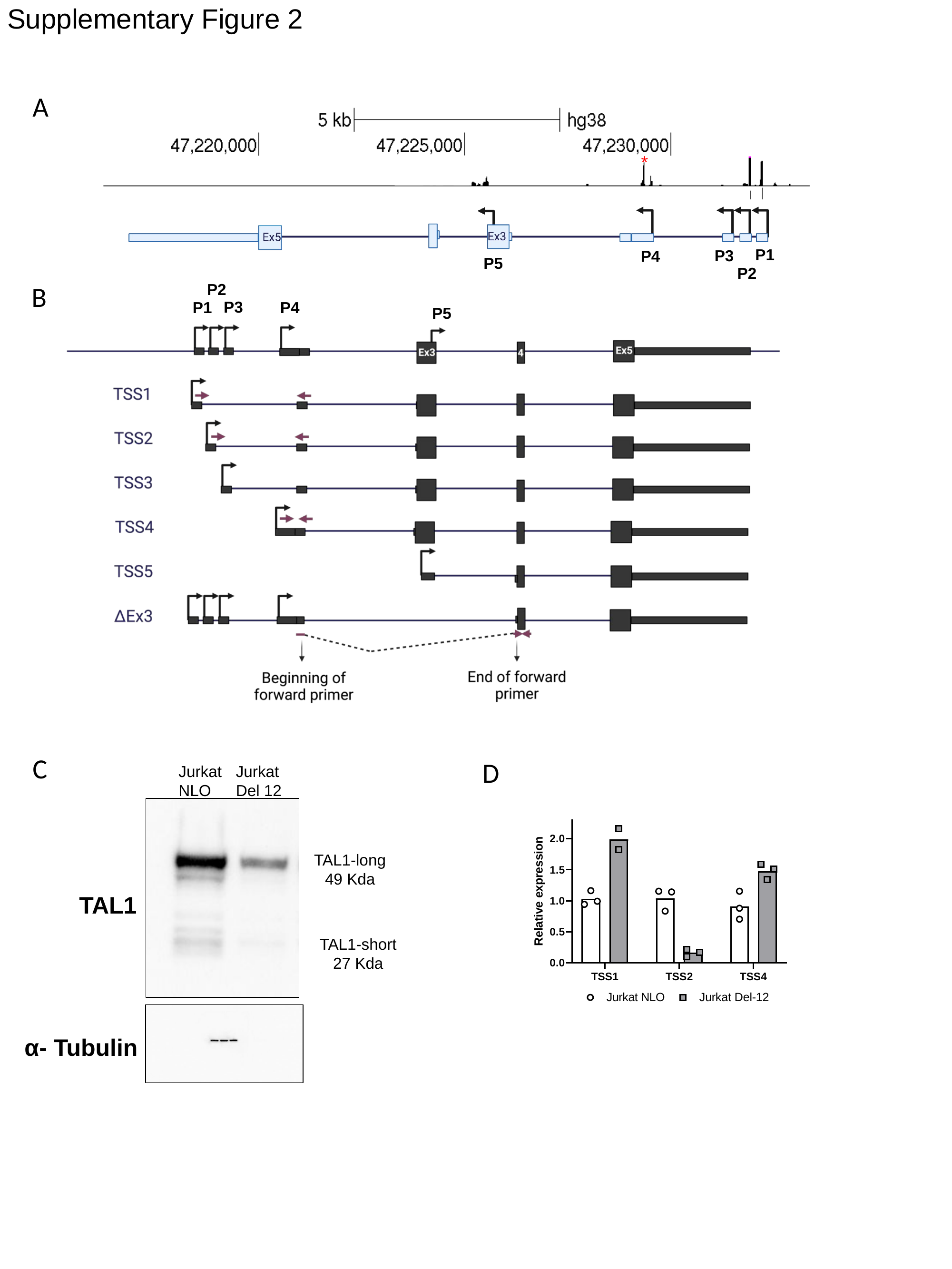

Supplementary Figure 2
A
*
P1
P3
P4
P5
P2
P2
B
P3
P1
P4
P5
C
D
Jurkat NLO
Jurkat Del 12
TAL1-long
49 Kda
TAL1
TAL1-short
27 Kda
α- Tubulin

## Slide 2
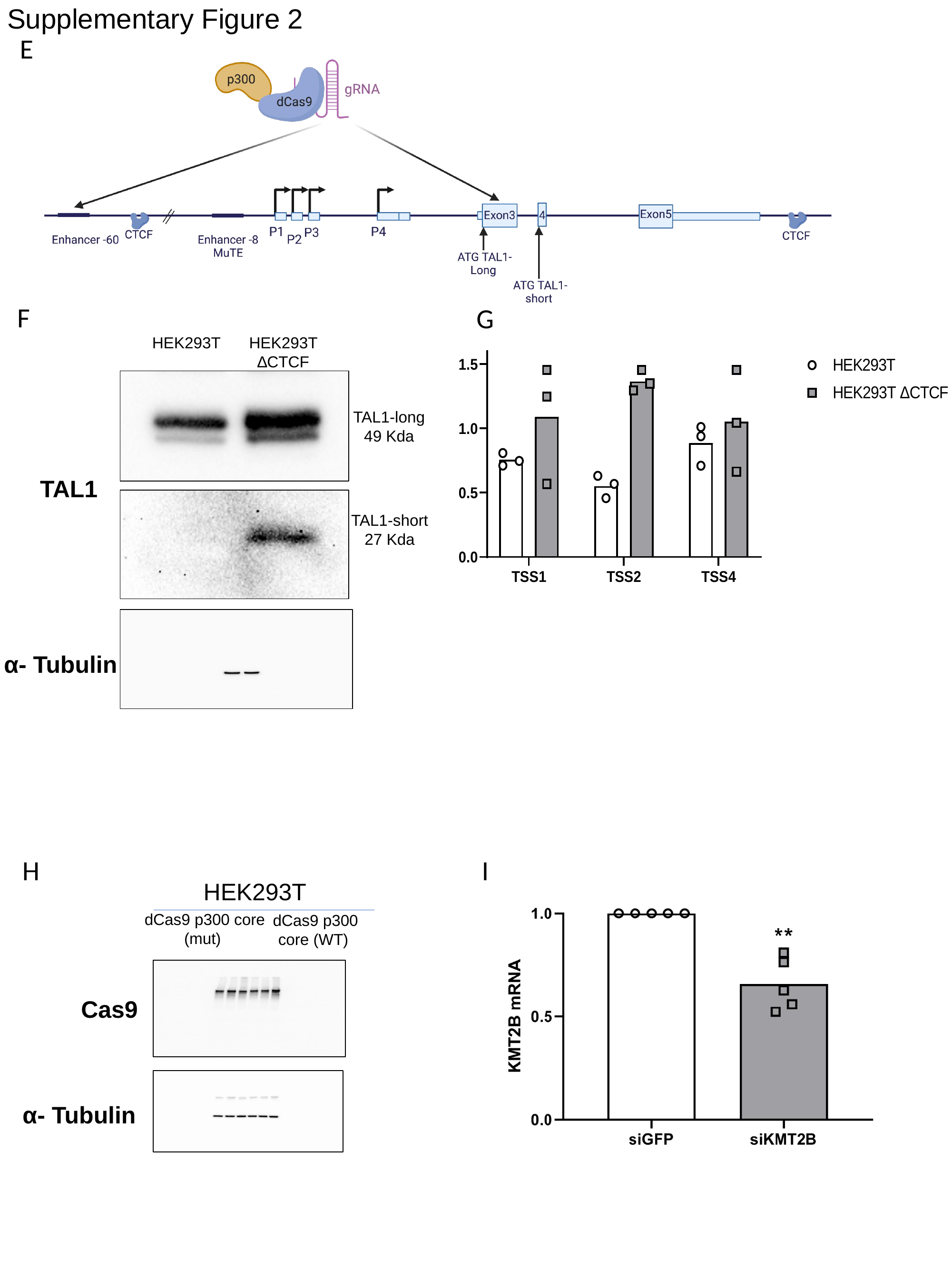

Supplementary Figure 2
E
F
G
HEK293T
HEK293T
∆CTCF
TAL1-long
49 Kda
TAL1-short
27 Kda
TAL1
α- Tubulin
H
I
HEK293T
dCas9 p300 core (mut)
dCas9 p300 core (WT)
Cas9
α- Tubulin

## Slide 3
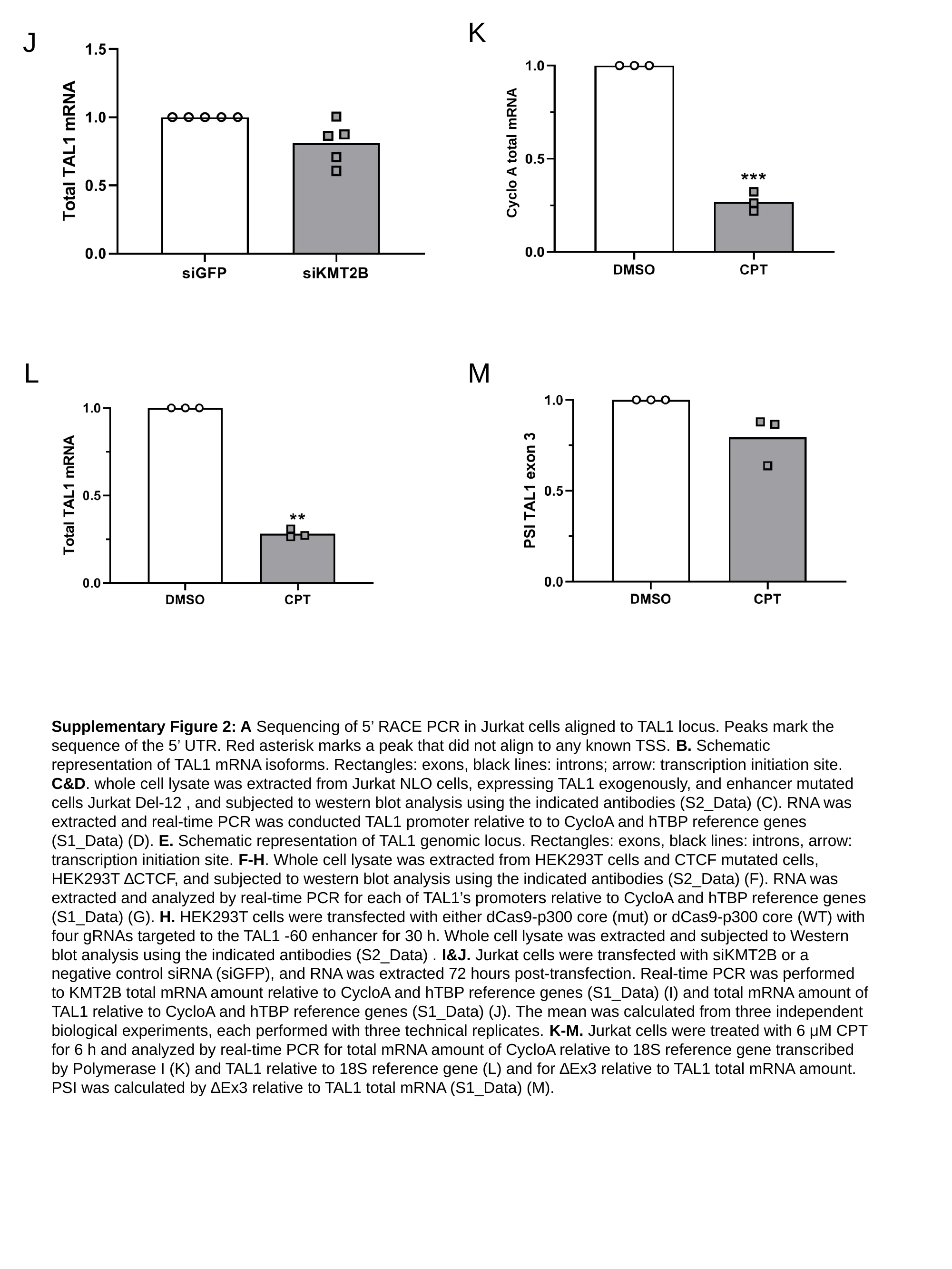

K
J
Cyclo A total mRNA
L
M
Supplementary Figure 2: A Sequencing of 5’ RACE PCR in Jurkat cells aligned to TAL1 locus. Peaks mark the sequence of the 5’ UTR. Red asterisk marks a peak that did not align to any known TSS. B. Schematic representation of TAL1 mRNA isoforms. Rectangles: exons, black lines: introns; arrow: transcription initiation site. C&D. whole cell lysate was extracted from Jurkat NLO cells, expressing TAL1 exogenously, and enhancer mutated cells Jurkat Del-12 , and subjected to western blot analysis using the indicated antibodies (S2_Data) (C). RNA was extracted and real-time PCR was conducted TAL1 promoter relative to to CycloA and hTBP reference genes (S1_Data) (D). E. Schematic representation of TAL1 genomic locus. Rectangles: exons, black lines: introns, arrow: transcription initiation site. F-H. Whole cell lysate was extracted from HEK293T cells and CTCF mutated cells, HEK293T ∆CTCF, and subjected to western blot analysis using the indicated antibodies (S2_Data) (F). RNA was extracted and analyzed by real-time PCR for each of TAL1’s promoters relative to CycloA and hTBP reference genes (S1_Data) (G). H. HEK293T cells were transfected with either dCas9-p300 core (mut) or dCas9-p300 core (WT) with four gRNAs targeted to the TAL1 -60 enhancer for 30 h. Whole cell lysate was extracted and subjected to Western blot analysis using the indicated antibodies (S2_Data) . I&J. Jurkat cells were transfected with siKMT2B or a negative control siRNA (siGFP), and RNA was extracted 72 hours post-transfection. Real-time PCR was performed to KMT2B total mRNA amount relative to CycloA and hTBP reference genes (S1_Data) (I) and total mRNA amount of TAL1 relative to CycloA and hTBP reference genes (S1_Data) (J). The mean was calculated from three independent biological experiments, each performed with three technical replicates. K-M. Jurkat cells were treated with 6 μM CPT for 6 h and analyzed by real-time PCR for total mRNA amount of CycloA relative to 18S reference gene transcribed by Polymerase I (K) and TAL1 relative to 18S reference gene (L) and for ∆Ex3 relative to TAL1 total mRNA amount. PSI was calculated by ∆Ex3 relative to TAL1 total mRNA (S1_Data) (M).
