# Supplementary material for: Isoforms of the TAL1 transcription factor have different roles in hematopoiesis and cell growth
Source: PLoS Biol. 2023 Jun 28;21(6):e3002175. doi: 10.1371/journal.pbio.3002175 (PMC10335695; doi:10.1371/journal.pbio.3002175)
Supplement: S3 Fig — (A-C) HEK293T cells were cotransfected with TAL1 promoters: promoters 1–3, 4, 5, and exon 4 as a negative control. The second plasmid was an empty vector, TAL1-short, or TAL1-long. After 30 h, RNA was extracted and real-time PCR was performed to TAL1 total mRNA amount relative to CycloA and hTBP reference genes (S1 Data) (A) and whole cell lysate was extracted and subjected to western blot analysis using the indicated antibodies (S1 Raw Images) (B) and RNA was extracted and analyzed by real-time PCR for luciferase mRNA relative to renilla mRNA (S1 Data) (C). (PPTX) [file pbio.3002175.s003.pptx]

## Slide 1
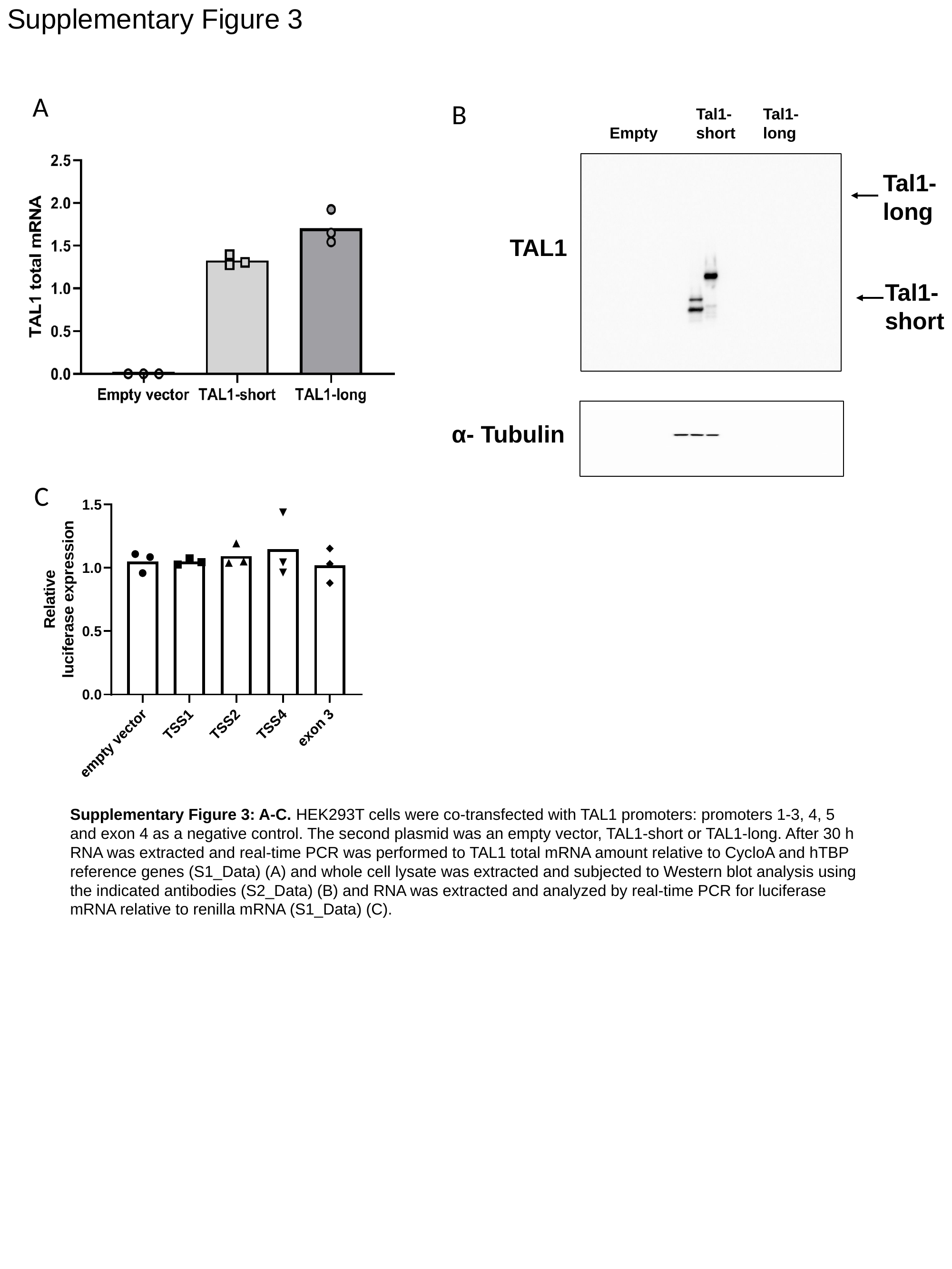

Supplementary Figure 3
Tal1-short
Tal1-long
A
B
Empty
Tal1-long
TAL1
Tal1- short
α- Tubulin
C
Supplementary Figure 3: A-C. HEK293T cells were co-transfected with TAL1 promoters: promoters 1-3, 4, 5 and exon 4 as a negative control. The second plasmid was an empty vector, TAL1-short or TAL1-long. After 30 h RNA was extracted and real-time PCR was performed to TAL1 total mRNA amount relative to CycloA and hTBP reference genes (S1_Data) (A) and whole cell lysate was extracted and subjected to Western blot analysis using the indicated antibodies (S2_Data) (B) and RNA was extracted and analyzed by real-time PCR for luciferase mRNA relative to renilla mRNA (S1_Data) (C).
