# Supplementary material for: Isoforms of the TAL1 transcription factor have different roles in hematopoiesis and cell growth
Source: PLoS Biol. 2023 Jun 28;21(6):e3002175. doi: 10.1371/journal.pbio.3002175 (PMC10335695; doi:10.1371/journal.pbio.3002175)
Supplement: S4 Fig — (A) Jurkat cells were infected with either empty vector, GFP-TAL1-short, or GFP-TAL1-long. GFP was immunoprecipitated and interacting proteins were detected with indicated antibodies (S1 Raw Images). (B-J) Jurkat cells were infected with either empty vector, FLAG-TAL1-short, or FLAG-TAL1-long. ChIP-seq was performed with anti-FLAG magnetic beads. In addition, we analyzed available data for TAL1-total (see methods). Whole cell lysate was extracted and subjected to western blot analysis using the indicated antibodies (S1 Raw Images) (B). ChIP-seq tracks are shown at the indicated locus for each biological experiment. Arrows indicate known TAL1-total binding site (C). ChIP-seq average signal for TAL1-total, TAL1-short and TAL1-long, empty vector and input as a function of distance from TAL1-total peaks (S1 Data) (D). ChIP-seq peaks across genomic regions (S1 Data) (E). Most abundant DNA sequence motifs identified in ChIP-seq peaks (F-J). (K-R) Jurkat cells were infected with shRNA to TAL1 3′ UTR and later with empty vector, FLAG-TAL1-short or FLAG-TAL1-long. Induction with tetracycline was conducted for 72 h, and silencing was measured using real-time PCR for total mRNA amount of endogenous TAL1 relative to CycloA and hTBP reference genes (S1 Data) (K). RNA-seq was preformed on 3 biological replicates and analyzed for isoforms specific targets. In addition, we analyzed available data for TAL1-total (see methods). Venn diagrams showing numbers of distinct and common RNA-seq targets and peak-associated genes of TAL1-total (L) and TAL1-short (M). Real-time PCR for total mRNA amount of 3 TAL1-short targets was performed relative to CycloA and hTBP reference genes (S1 Data) (N). Bar chart of top 4 enriched terms from the GO_Biological_Process_2018 gene set library for TAL1-total (O) and TAL1-short (P) and TAL1-long (Q). Heatmap and statistics for expression level of apoptosis pathway genes from GSEA plot (R). (PPTX) [file pbio.3002175.s004.pptx]

## Slide 1
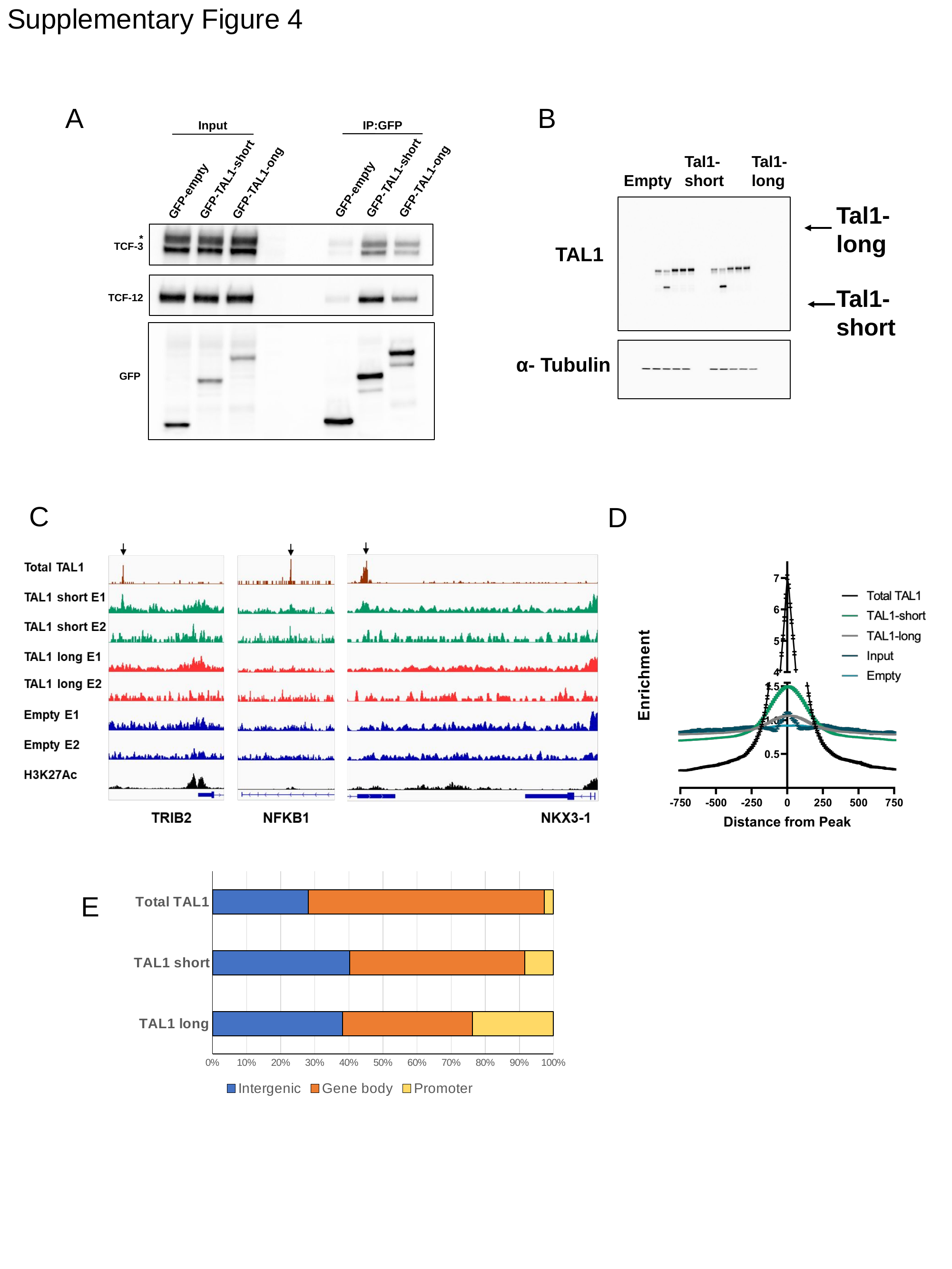

Supplementary Figure 4
GFP-empty
GFP-TAL1-ong
GFP-TAL1-short
GFP-empty
GFP-TAL1-ong
GFP-TAL1-short
Input
IP:GFP
*
TCF-3
TCF-12
GFP
A
B
Tal1-short
Tal1-long
Empty
Tal1-long
TAL1
Tal1- short
α- Tubulin
C
D
### Chart
| Category | Intergenic | Gene body | Promoter |
|---|---|---|---|
| TAL1 long | 8.0 | 8.0 | 5.0 |
| TAL1 short | 312.0 | 399.0 | 65.0 |
| Total TAL1 | 2271.0 | 5602.0 | 221.0 |E

## Slide 2
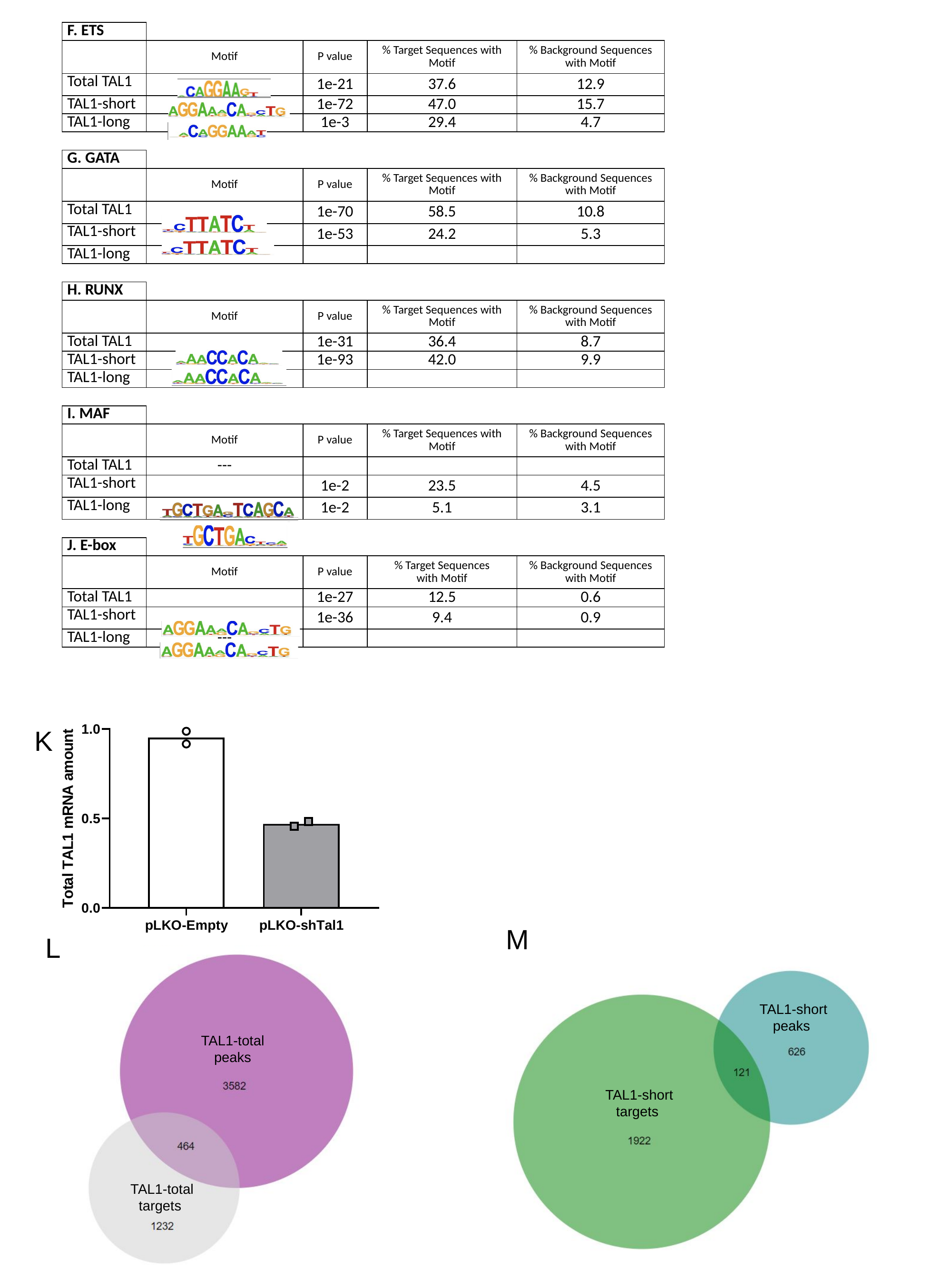

| F. ETS | | | | |
| --- | --- | --- | --- | --- |
| | Motif | P value | % Target Sequences with Motif | % Background Sequences with Motif |
| Total TAL1 | | 1e-21 | 37.6 | 12.9 |
| TAL1-short | | 1e-72 | 47.0 | 15.7 |
| TAL1-long | | 1e-3 | 29.4 | 4.7 |
| | | | | |
| G. GATA | | | | |
| | Motif | P value | % Target Sequences with Motif | % Background Sequences with Motif |
| Total TAL1 | | 1e-70 | 58.5 | 10.8 |
| TAL1-short | | 1e-53 | 24.2 | 5.3 |
| TAL1-long | --- | | | |
| | | | | |
| H. RUNX | | | | |
| | Motif | P value | % Target Sequences with Motif | % Background Sequences with Motif |
| Total TAL1 | | 1e-31 | 36.4 | 8.7 |
| TAL1-short | | 1e-93 | 42.0 | 9.9 |
| TAL1-long | --- | | | |
| | | | | |
| I. MAF | | | | |
| | Motif | P value | % Target Sequences with Motif | % Background Sequences with Motif |
| Total TAL1 | --- | | | |
| TAL1-short | | 1e-2 | 23.5 | 4.5 |
| TAL1-long | | 1e-2 | 5.1 | 3.1 |
| | | | | |
| J. E-box | | | | |
| | Motif | P value | % Target Sequences with Motif | % Background Sequences with Motif |
| Total TAL1 | | 1e-27 | 12.5 | 0.6 |
| TAL1-short | | 1e-36 | 9.4 | 0.9 |
| TAL1-long | --- | | | |
K
M
L
TAL1-total peaks
TAL1-total targets
TAL1-short peaks
TAL1-short targets

## Slide 3
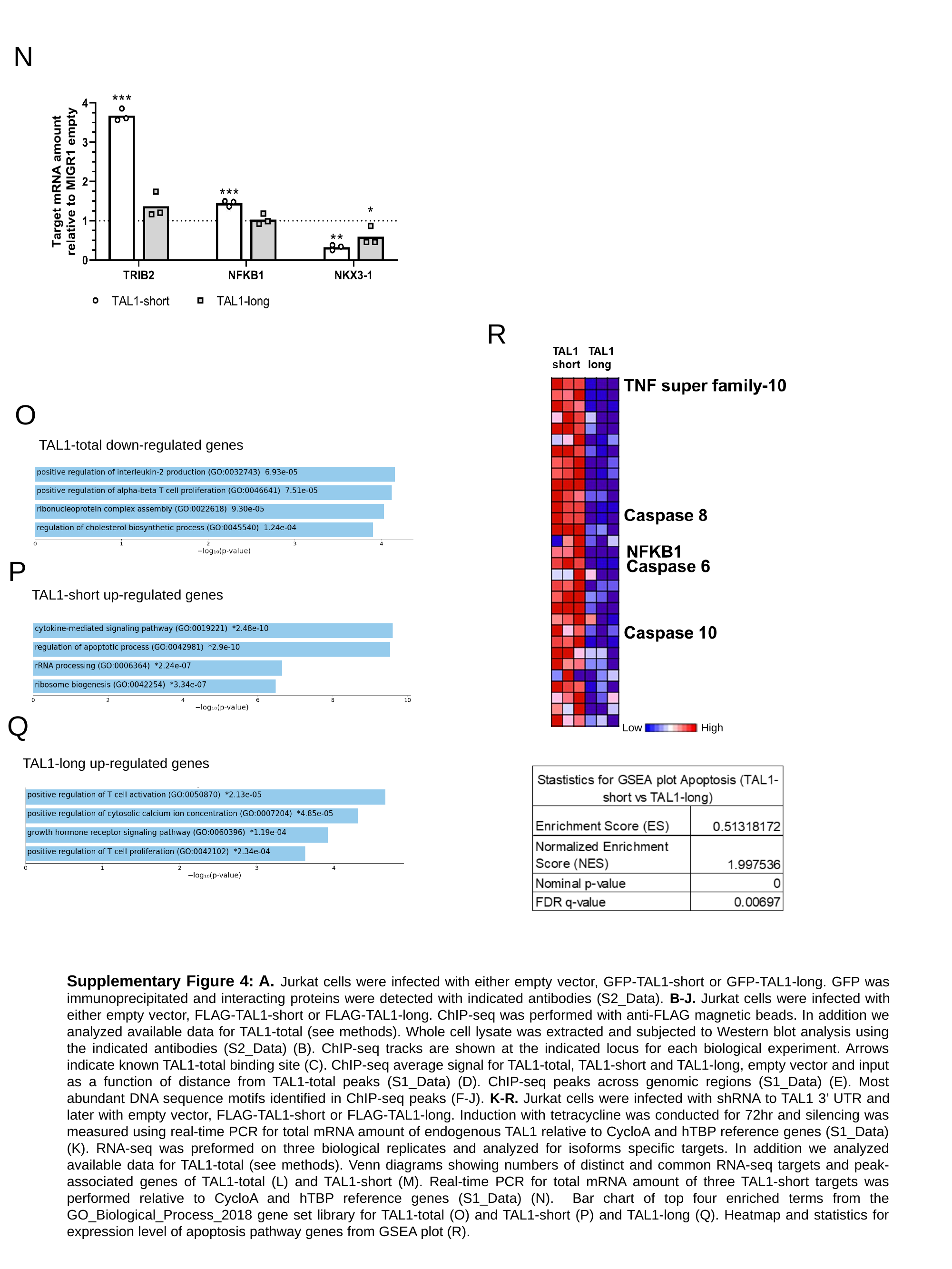

N
R
Low
High
O
TAL1-total down-regulated genes
P
TAL1-short up-regulated genes
Q
TAL1-long up-regulated genes
Supplementary Figure 4: A. Jurkat cells were infected with either empty vector, GFP-TAL1-short or GFP-TAL1-long. GFP was immunoprecipitated and interacting proteins were detected with indicated antibodies (S2_Data). B-J. Jurkat cells were infected with either empty vector, FLAG-TAL1-short or FLAG-TAL1-long. ChIP-seq was performed with anti-FLAG magnetic beads. In addition we analyzed available data for TAL1-total (see methods). Whole cell lysate was extracted and subjected to Western blot analysis using the indicated antibodies (S2_Data) (B). ChIP-seq tracks are shown at the indicated locus for each biological experiment. Arrows indicate known TAL1-total binding site (C). ChIP-seq average signal for TAL1-total, TAL1-short and TAL1-long, empty vector and input as a function of distance from TAL1-total peaks (S1_Data) (D). ChIP-seq peaks across genomic regions (S1_Data) (E). Most abundant DNA sequence motifs identified in ChIP-seq peaks (F-J). K-R. Jurkat cells were infected with shRNA to TAL1 3’ UTR and later with empty vector, FLAG-TAL1-short or FLAG-TAL1-long. Induction with tetracycline was conducted for 72hr and silencing was measured using real-time PCR for total mRNA amount of endogenous TAL1 relative to CycloA and hTBP reference genes (S1_Data) (K). RNA-seq was preformed on three biological replicates and analyzed for isoforms specific targets. In addition we analyzed available data for TAL1-total (see methods). Venn diagrams showing numbers of distinct and common RNA-seq targets and peak-associated genes of TAL1-total (L) and TAL1-short (M). Real-time PCR for total mRNA amount of three TAL1-short targets was performed relative to CycloA and hTBP reference genes (S1_Data) (N). Bar chart of top four enriched terms from the GO_Biological_Process_2018 gene set library for TAL1-total (O) and TAL1-short (P) and TAL1-long (Q). Heatmap and statistics for expression level of apoptosis pathway genes from GSEA plot (R).
