# Supplementary material for: Isoforms of the TAL1 transcription factor have different roles in hematopoiesis and cell growth
Source: PLoS Biol. 2023 Jun 28;21(6):e3002175. doi: 10.1371/journal.pbio.3002175 (PMC10335695; doi:10.1371/journal.pbio.3002175)
Supplement: S5 Fig — (A-K) Equal numbers of bone marrow cells from 5FU-treated CD45.1 wild-type mice were transduced with retroviruses expressing either TAL1-short-GFP or with TAL1-long-dtTomato. A mixture of 1:1 ratio of the transduced bone marrow cells was then transplanted into lethally irradiated CD45.2 wild-type recipient mice. Mice were killed 14 weeks after BMT, and the bone marrow cells were harvested and analyzed using flow cytometric analysis. (A) Representative flow cytometry dot plots of Ly6C vs. Ly6G staining gated on CD11b-positive cells that are GFP−/dtTomato− (untransduced, left panel), dtTomato+ (Tal1-long, middle panel) or GFP+ (Tal1-short, right panel) splenocytes. Regions represent the following populations: R1− CD11b+, Ly6C−, Ly6G− (circulating monocytes), R2− CD11b+, Ly6Chi, Ly6G− (inflammatory monocytes), R3− CD11b+, Ly6Cint, Ly6G+ (granulocytes). (B) Bar graph summarizing results in c (S1 Data). (C) Representative flow cytometry dot plots analysis of CD71 and Ter119 staining gated on CD45-negative cells that are GFP−/dtTomato− (untransduced, left panel), dtTomato+ (Tal1-long, middle panel), or GFP+ (Tal1-short, right panel). Regions S0-S5 represent the stage in erythrocyte differentiation. (D) Bar graph summarizing results in e (S1 Data). Left panel: percentages of CD71− Ter119− cells (the S0 phase in erythrocytes differentiation); right panel: CD71+ Ter119hi (the S3 phase in erythrocytes differentiation. (E) Frequencies of the subpopulation of erythrocytes in the different differentiation stages based on the CD71 and Ter119 expression (S1 Data). (F) Representative flow cytometry dot plots of Thy1.2 vs. CD19 staining gated on GFP−/dtTomato− (untransduced, left panel), dtTomato+ (Tal1-long, middle panel), or GFP+ (Tal1-short, right panel) bone marrow cells. (G) Bar graph summarizing results in a (S1 Data). (H) Representative flow cytometry histograms of CD11b staining gated on GFP−/dtTomato− (untransduced, left panel), dtTomato+ (Tal1-long, middle panel), or GFP+ [file pbio.3002175.s005.pptx]

## Slide 1
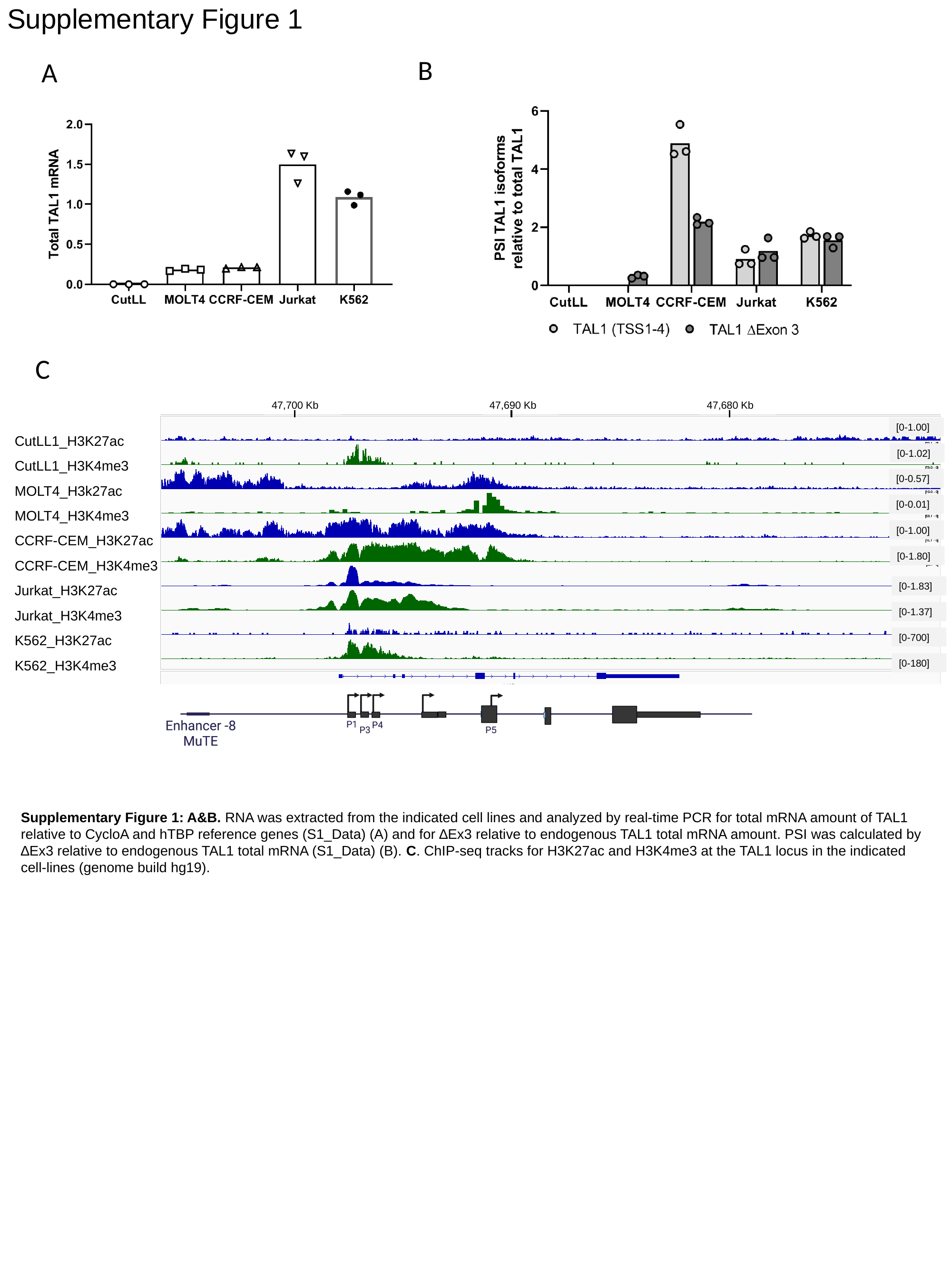

Supplementary Figure 1
B
A
C
47,700 Kb
47,690 Kb
47,680 Kb
[0-1.00]
CutLL1_H3K27ac
CutLL1_H3K4me3
MOLT4_H3k27ac
MOLT4_H3K4me3
CCRF-CEM_H3K27ac
CCRF-CEM_H3K4me3
Jurkat_H3K27ac
Jurkat_H3K4me3
K562_H3K27ac
K562_H3K4me3
[0-1.02]
[0-0.57]
[0-0.01]
[0-1.00]
[0-1.80]
[0-700]
[0-180]
[0-1.83]
[0-1.37]
Supplementary Figure 1: A&B. RNA was extracted from the indicated cell lines and analyzed by real-time PCR for total mRNA amount of TAL1 relative to CycloA and hTBP reference genes (S1_Data) (A) and for ∆Ex3 relative to endogenous TAL1 total mRNA amount. PSI was calculated by ∆Ex3 relative to endogenous TAL1 total mRNA (S1_Data) (B). C. ChIP-seq tracks for H3K27ac and H3K4me3 at the TAL1 locus in the indicated cell-lines (genome build hg19).

## Slide 2
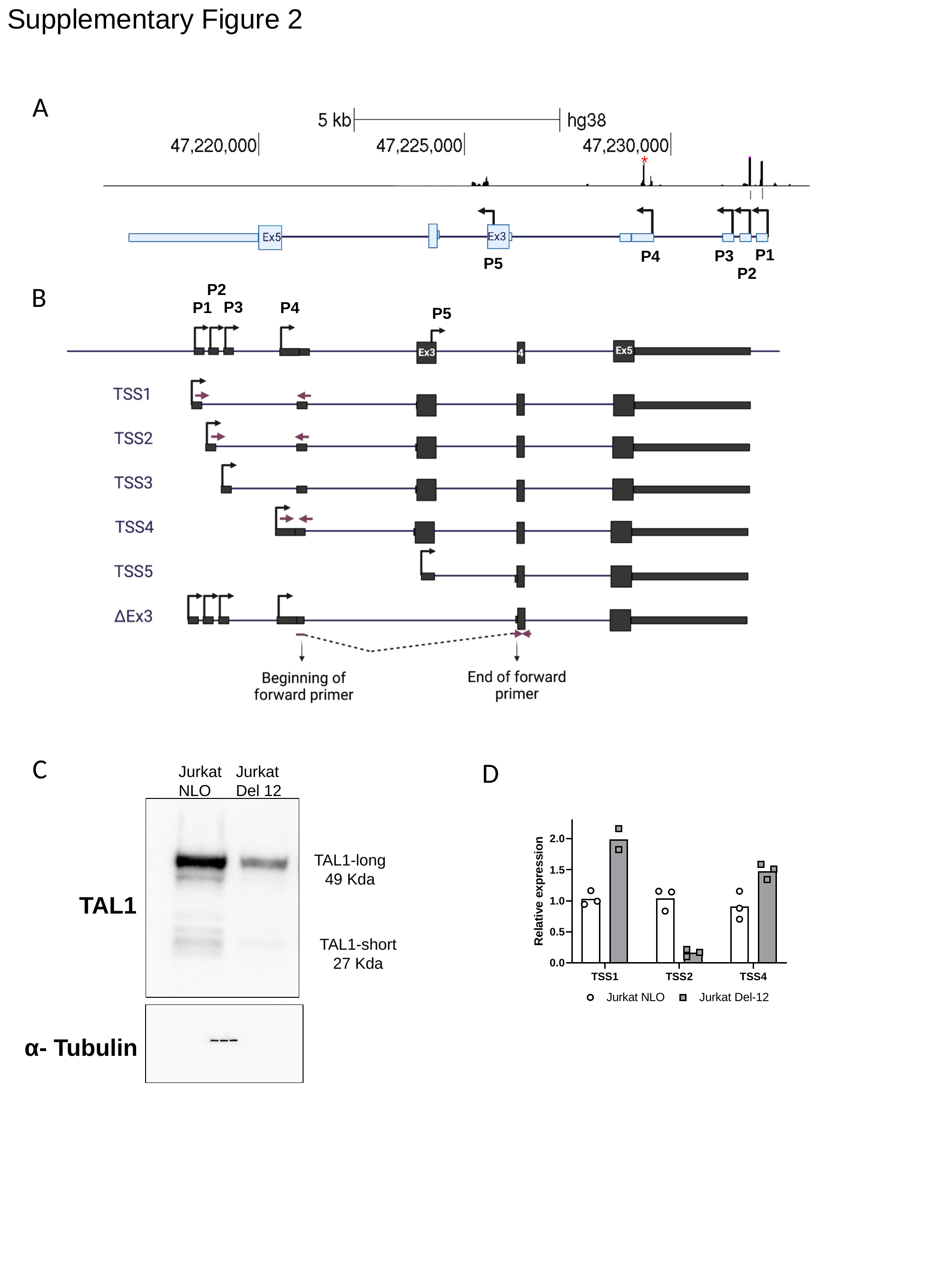

Supplementary Figure 2
A
*
P1
P3
P4
P5
P2
P2
B
P3
P1
P4
P5
C
D
Jurkat NLO
Jurkat Del 12
TAL1-long
49 Kda
TAL1
TAL1-short
27 Kda
α- Tubulin

## Slide 3
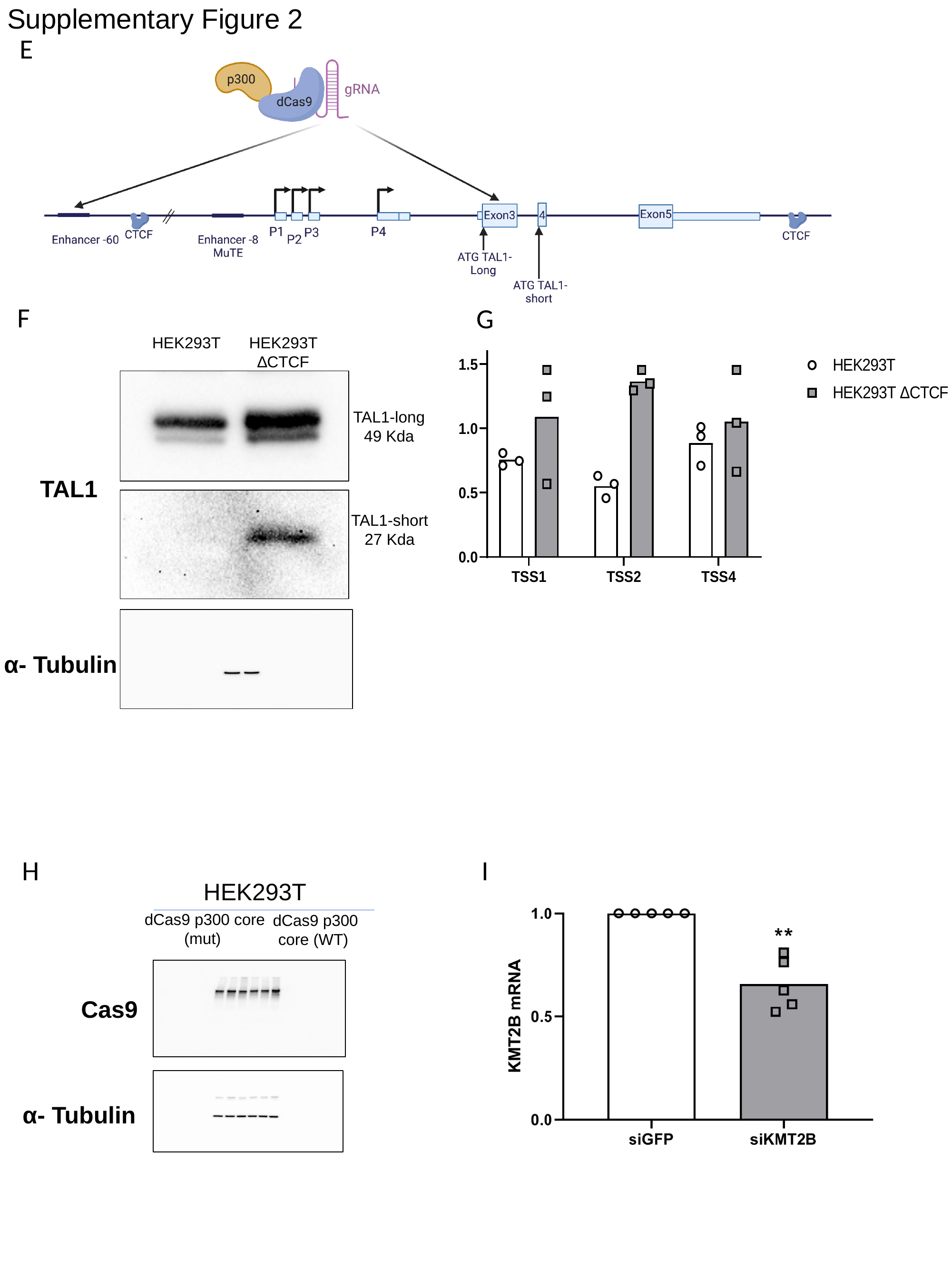

Supplementary Figure 2
E
F
G
HEK293T
HEK293T
∆CTCF
TAL1-long
49 Kda
TAL1-short
27 Kda
TAL1
α- Tubulin
H
I
HEK293T
dCas9 p300 core (mut)
dCas9 p300 core (WT)
Cas9
α- Tubulin

## Slide 4
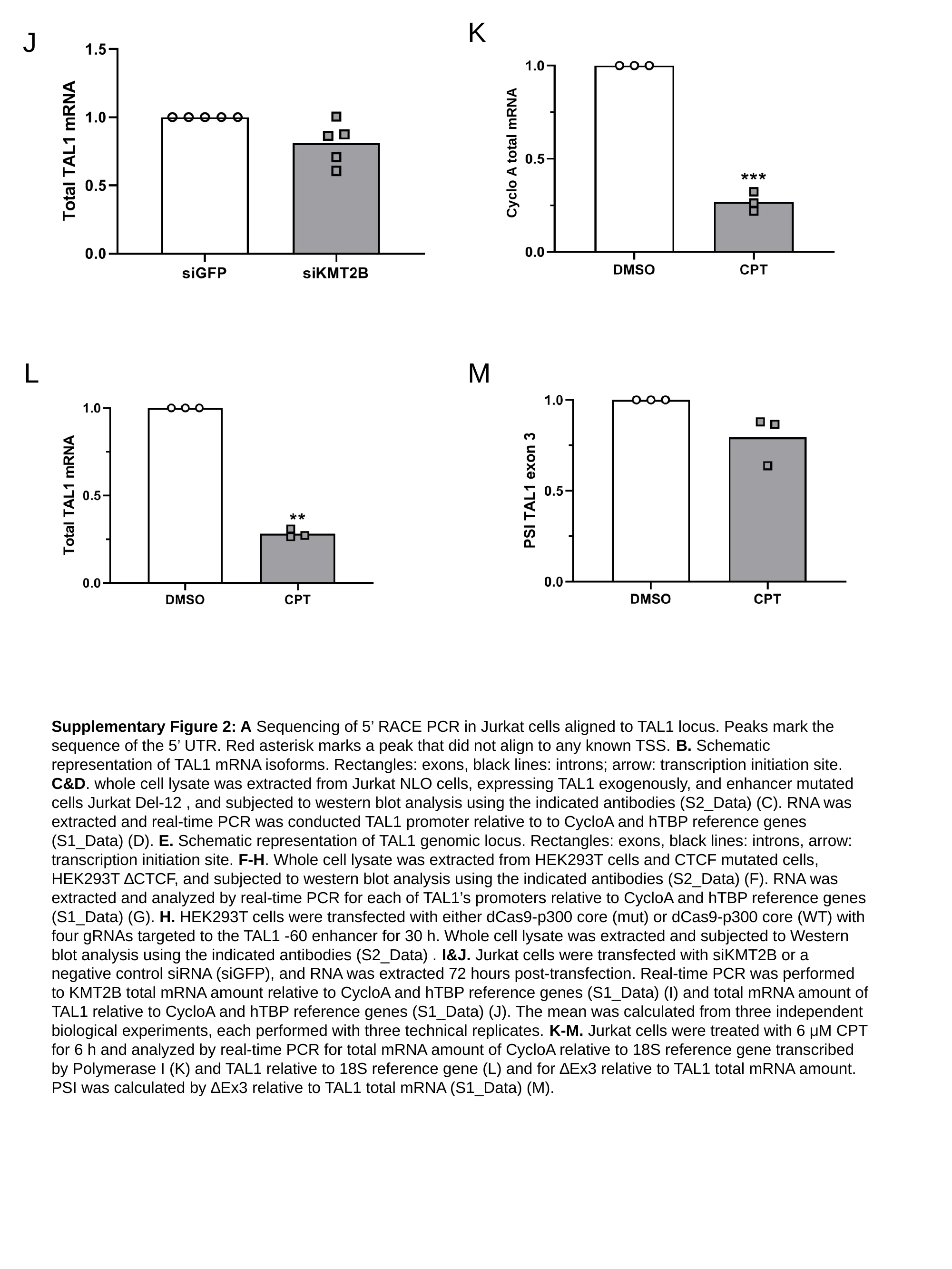

K
J
Cyclo A total mRNA
L
M
Supplementary Figure 2: A Sequencing of 5’ RACE PCR in Jurkat cells aligned to TAL1 locus. Peaks mark the sequence of the 5’ UTR. Red asterisk marks a peak that did not align to any known TSS. B. Schematic representation of TAL1 mRNA isoforms. Rectangles: exons, black lines: introns; arrow: transcription initiation site. C&D. whole cell lysate was extracted from Jurkat NLO cells, expressing TAL1 exogenously, and enhancer mutated cells Jurkat Del-12 , and subjected to western blot analysis using the indicated antibodies (S2_Data) (C). RNA was extracted and real-time PCR was conducted TAL1 promoter relative to to CycloA and hTBP reference genes (S1_Data) (D). E. Schematic representation of TAL1 genomic locus. Rectangles: exons, black lines: introns, arrow: transcription initiation site. F-H. Whole cell lysate was extracted from HEK293T cells and CTCF mutated cells, HEK293T ∆CTCF, and subjected to western blot analysis using the indicated antibodies (S2_Data) (F). RNA was extracted and analyzed by real-time PCR for each of TAL1’s promoters relative to CycloA and hTBP reference genes (S1_Data) (G). H. HEK293T cells were transfected with either dCas9-p300 core (mut) or dCas9-p300 core (WT) with four gRNAs targeted to the TAL1 -60 enhancer for 30 h. Whole cell lysate was extracted and subjected to Western blot analysis using the indicated antibodies (S2_Data) . I&J. Jurkat cells were transfected with siKMT2B or a negative control siRNA (siGFP), and RNA was extracted 72 hours post-transfection. Real-time PCR was performed to KMT2B total mRNA amount relative to CycloA and hTBP reference genes (S1_Data) (I) and total mRNA amount of TAL1 relative to CycloA and hTBP reference genes (S1_Data) (J). The mean was calculated from three independent biological experiments, each performed with three technical replicates. K-M. Jurkat cells were treated with 6 μM CPT for 6 h and analyzed by real-time PCR for total mRNA amount of CycloA relative to 18S reference gene transcribed by Polymerase I (K) and TAL1 relative to 18S reference gene (L) and for ∆Ex3 relative to TAL1 total mRNA amount. PSI was calculated by ∆Ex3 relative to TAL1 total mRNA (S1_Data) (M).

## Slide 5
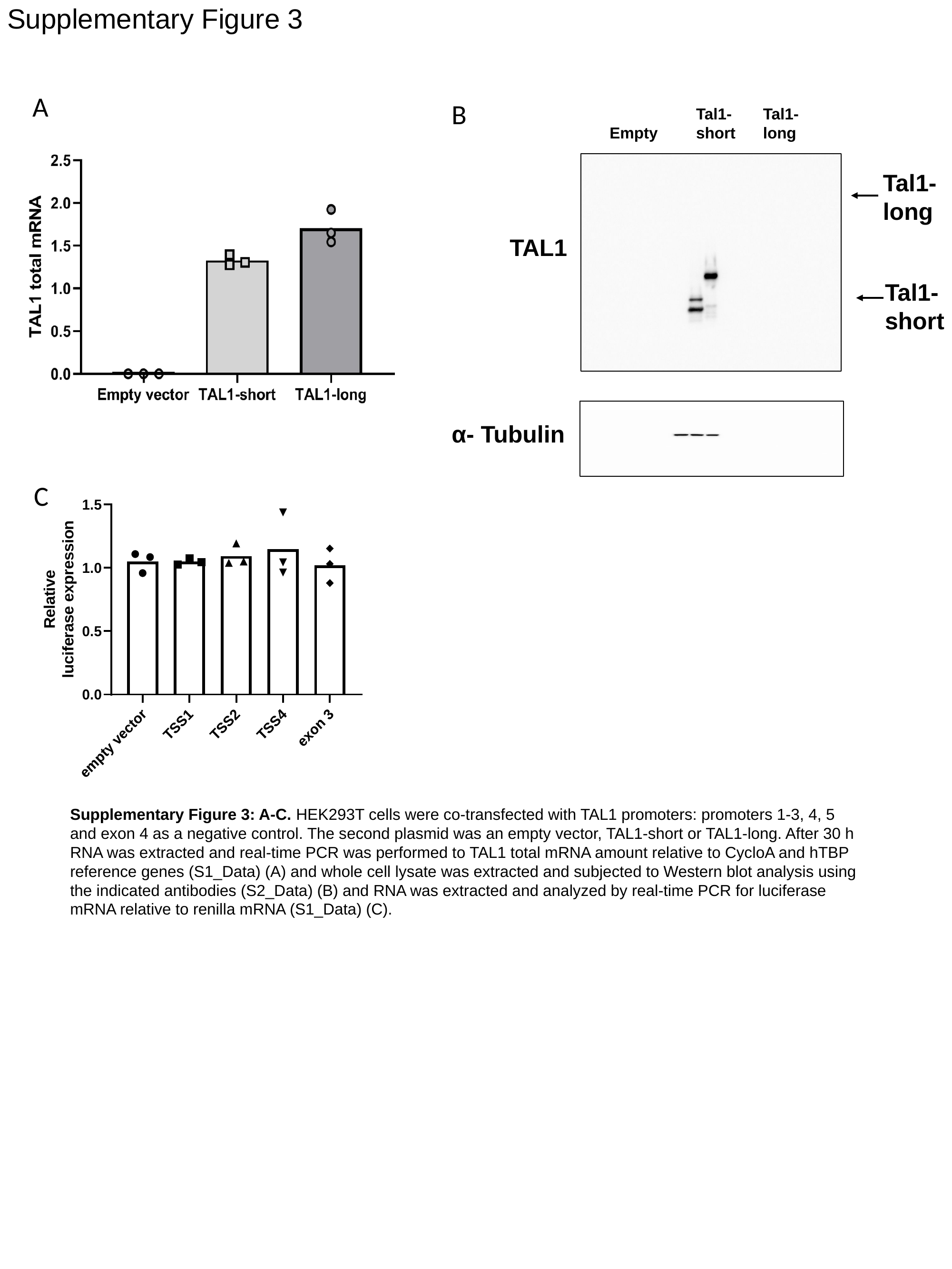

Supplementary Figure 3
Tal1-short
Tal1-long
A
B
Empty
Tal1-long
TAL1
Tal1- short
α- Tubulin
C
Supplementary Figure 3: A-C. HEK293T cells were co-transfected with TAL1 promoters: promoters 1-3, 4, 5 and exon 4 as a negative control. The second plasmid was an empty vector, TAL1-short or TAL1-long. After 30 h RNA was extracted and real-time PCR was performed to TAL1 total mRNA amount relative to CycloA and hTBP reference genes (S1_Data) (A) and whole cell lysate was extracted and subjected to Western blot analysis using the indicated antibodies (S2_Data) (B) and RNA was extracted and analyzed by real-time PCR for luciferase mRNA relative to renilla mRNA (S1_Data) (C).

## Slide 6
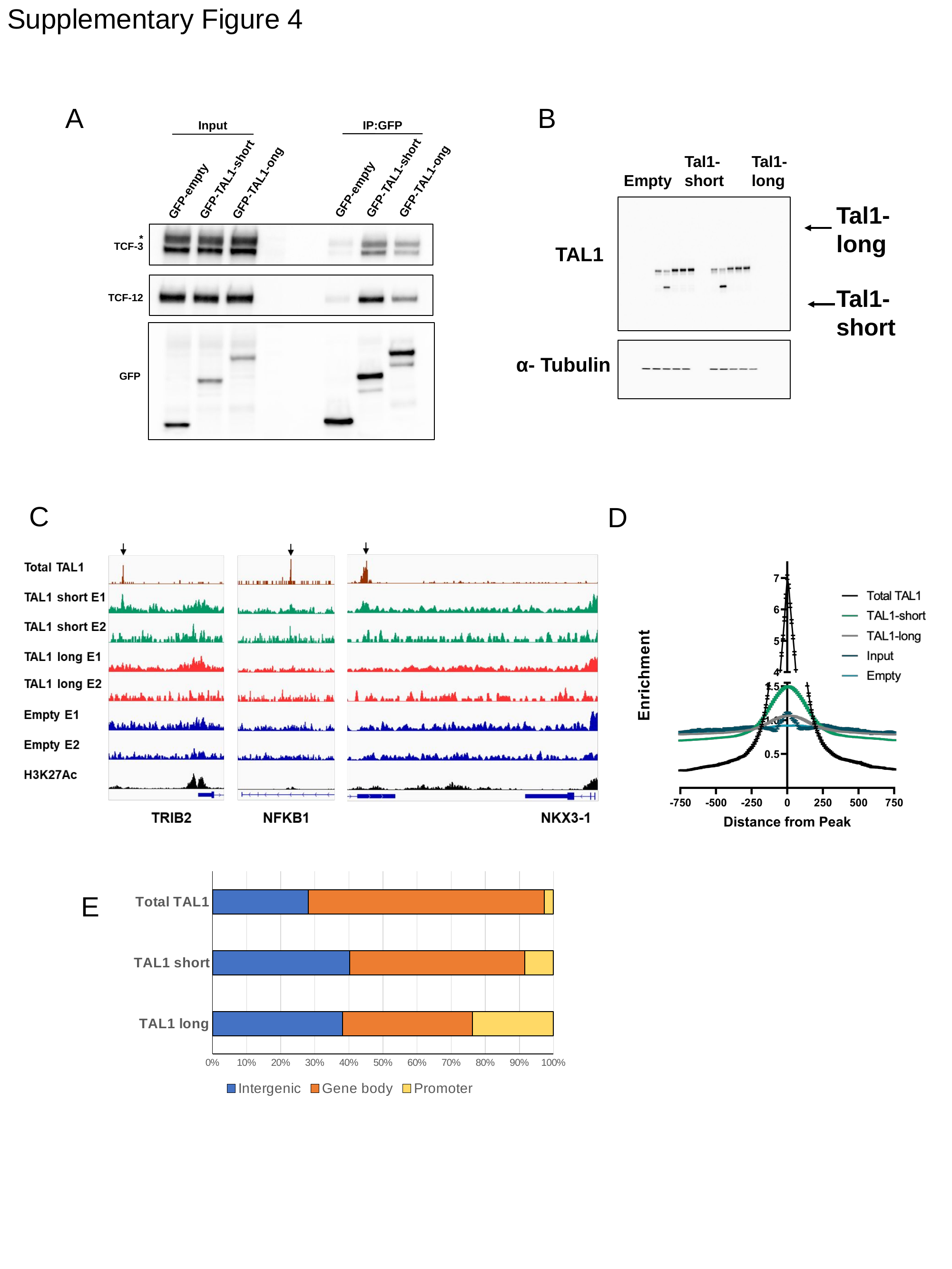

Supplementary Figure 4
GFP-empty
GFP-TAL1-ong
GFP-TAL1-short
GFP-empty
GFP-TAL1-ong
GFP-TAL1-short
Input
IP:GFP
*
TCF-3
TCF-12
GFP
A
B
Tal1-short
Tal1-long
Empty
Tal1-long
TAL1
Tal1- short
α- Tubulin
C
D
### Chart
| Category | Intergenic | Gene body | Promoter |
|---|---|---|---|
| TAL1 long | 8.0 | 8.0 | 5.0 |
| TAL1 short | 312.0 | 399.0 | 65.0 |
| Total TAL1 | 2271.0 | 5602.0 | 221.0 |E

## Slide 7
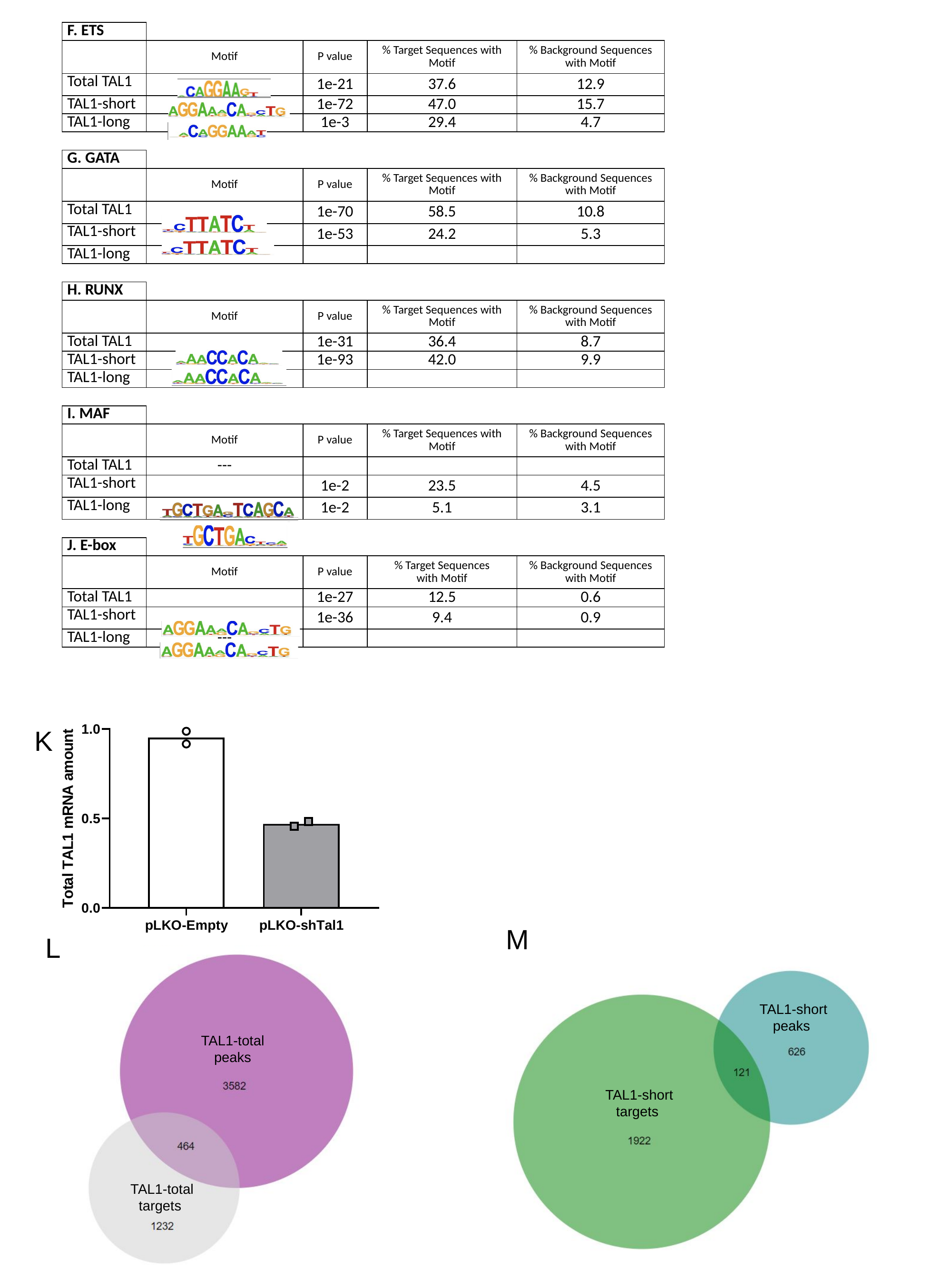

| F. ETS | | | | |
| --- | --- | --- | --- | --- |
| | Motif | P value | % Target Sequences with Motif | % Background Sequences with Motif |
| Total TAL1 | | 1e-21 | 37.6 | 12.9 |
| TAL1-short | | 1e-72 | 47.0 | 15.7 |
| TAL1-long | | 1e-3 | 29.4 | 4.7 |
| | | | | |
| G. GATA | | | | |
| | Motif | P value | % Target Sequences with Motif | % Background Sequences with Motif |
| Total TAL1 | | 1e-70 | 58.5 | 10.8 |
| TAL1-short | | 1e-53 | 24.2 | 5.3 |
| TAL1-long | --- | | | |
| | | | | |
| H. RUNX | | | | |
| | Motif | P value | % Target Sequences with Motif | % Background Sequences with Motif |
| Total TAL1 | | 1e-31 | 36.4 | 8.7 |
| TAL1-short | | 1e-93 | 42.0 | 9.9 |
| TAL1-long | --- | | | |
| | | | | |
| I. MAF | | | | |
| | Motif | P value | % Target Sequences with Motif | % Background Sequences with Motif |
| Total TAL1 | --- | | | |
| TAL1-short | | 1e-2 | 23.5 | 4.5 |
| TAL1-long | | 1e-2 | 5.1 | 3.1 |
| | | | | |
| J. E-box | | | | |
| | Motif | P value | % Target Sequences with Motif | % Background Sequences with Motif |
| Total TAL1 | | 1e-27 | 12.5 | 0.6 |
| TAL1-short | | 1e-36 | 9.4 | 0.9 |
| TAL1-long | --- | | | |
K
M
L
TAL1-total peaks
TAL1-total targets
TAL1-short peaks
TAL1-short targets

## Slide 8
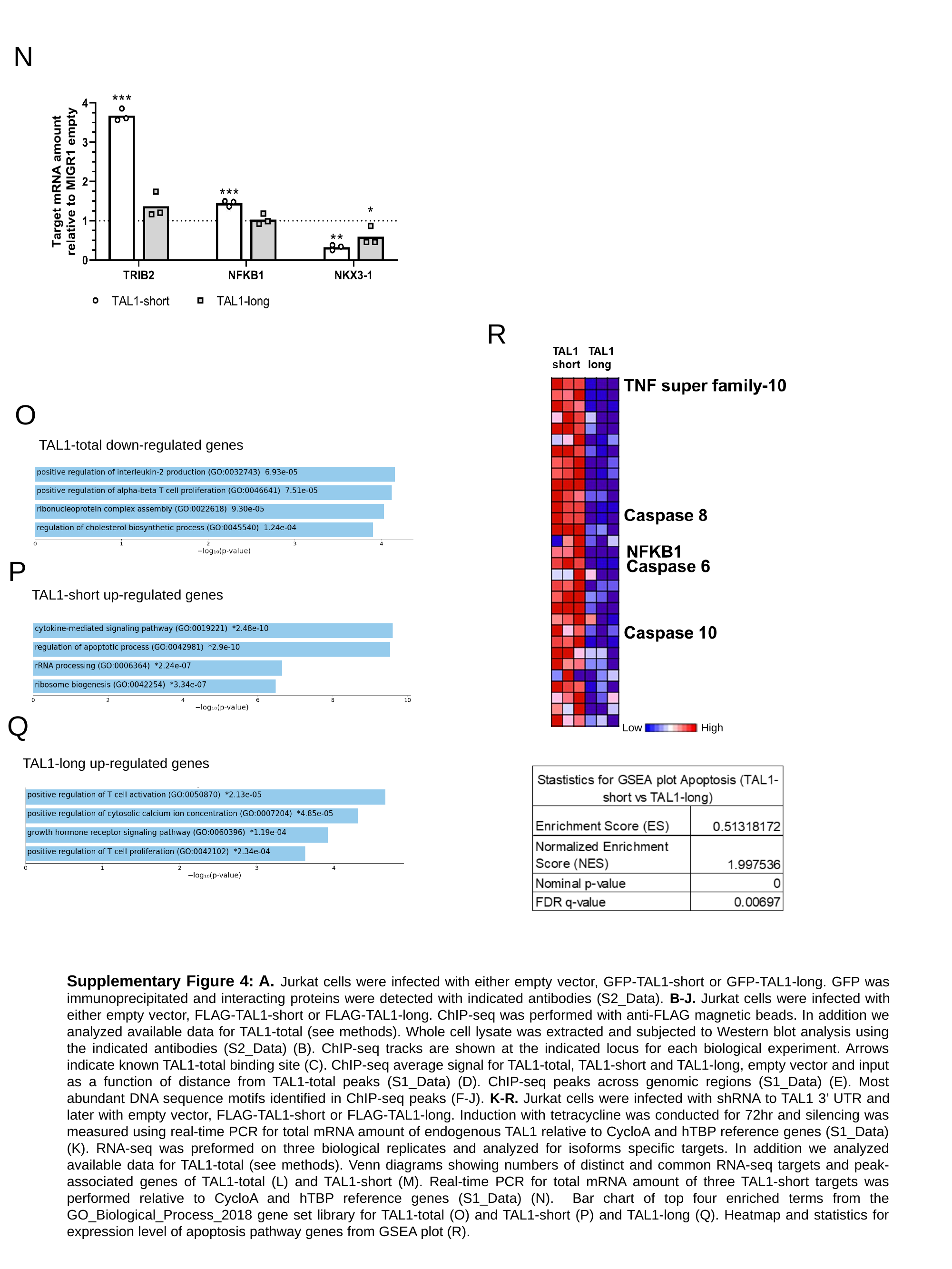

N
R
Low
High
O
TAL1-total down-regulated genes
P
TAL1-short up-regulated genes
Q
TAL1-long up-regulated genes
Supplementary Figure 4: A. Jurkat cells were infected with either empty vector, GFP-TAL1-short or GFP-TAL1-long. GFP was immunoprecipitated and interacting proteins were detected with indicated antibodies (S2_Data). B-J. Jurkat cells were infected with either empty vector, FLAG-TAL1-short or FLAG-TAL1-long. ChIP-seq was performed with anti-FLAG magnetic beads. In addition we analyzed available data for TAL1-total (see methods). Whole cell lysate was extracted and subjected to Western blot analysis using the indicated antibodies (S2_Data) (B). ChIP-seq tracks are shown at the indicated locus for each biological experiment. Arrows indicate known TAL1-total binding site (C). ChIP-seq average signal for TAL1-total, TAL1-short and TAL1-long, empty vector and input as a function of distance from TAL1-total peaks (S1_Data) (D). ChIP-seq peaks across genomic regions (S1_Data) (E). Most abundant DNA sequence motifs identified in ChIP-seq peaks (F-J). K-R. Jurkat cells were infected with shRNA to TAL1 3’ UTR and later with empty vector, FLAG-TAL1-short or FLAG-TAL1-long. Induction with tetracycline was conducted for 72hr and silencing was measured using real-time PCR for total mRNA amount of endogenous TAL1 relative to CycloA and hTBP reference genes (S1_Data) (K). RNA-seq was preformed on three biological replicates and analyzed for isoforms specific targets. In addition we analyzed available data for TAL1-total (see methods). Venn diagrams showing numbers of distinct and common RNA-seq targets and peak-associated genes of TAL1-total (L) and TAL1-short (M). Real-time PCR for total mRNA amount of three TAL1-short targets was performed relative to CycloA and hTBP reference genes (S1_Data) (N). Bar chart of top four enriched terms from the GO_Biological_Process_2018 gene set library for TAL1-total (O) and TAL1-short (P) and TAL1-long (Q). Heatmap and statistics for expression level of apoptosis pathway genes from GSEA plot (R).

## Slide 9
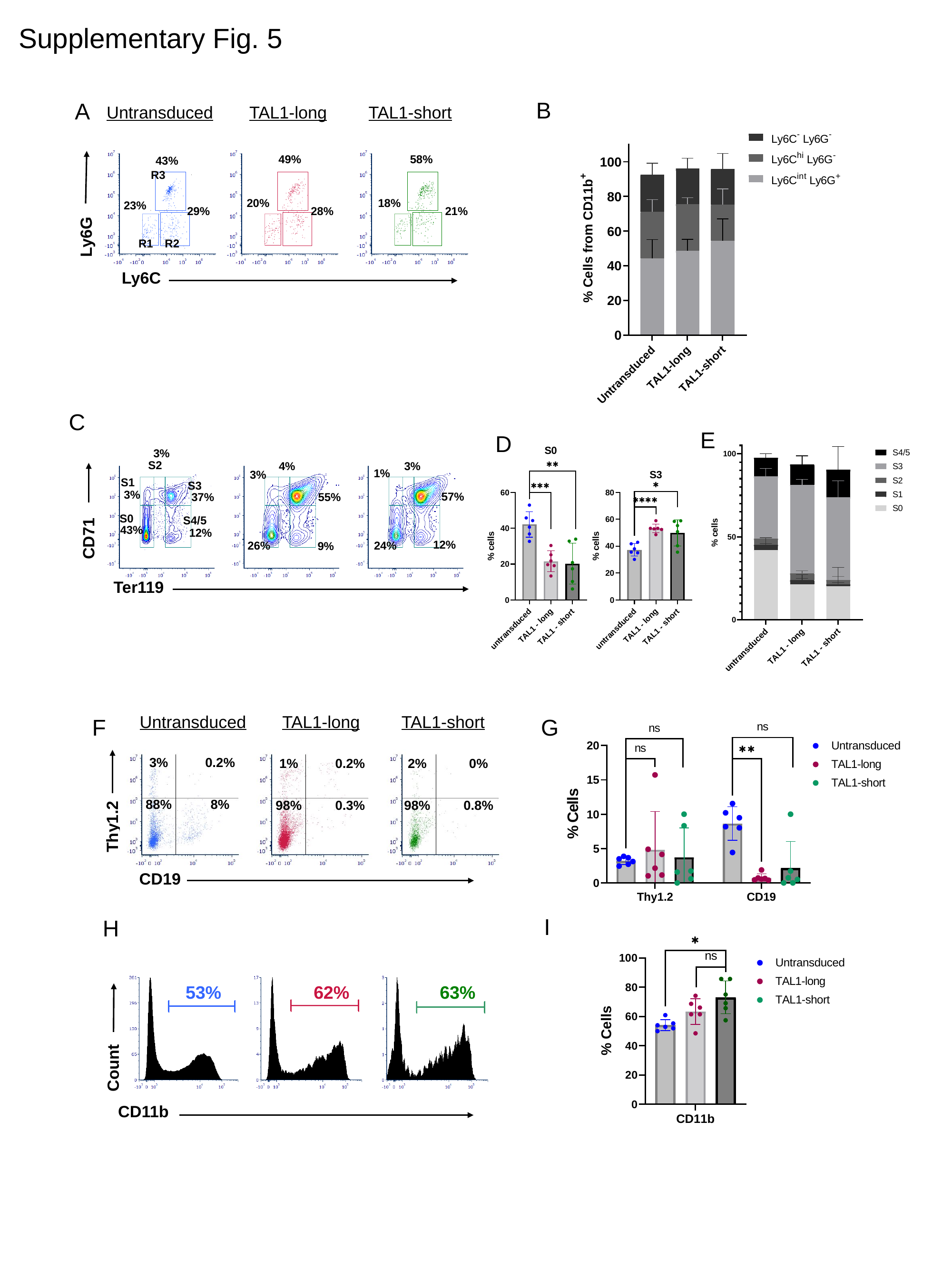

Supplementary Fig. 5
B
A
Untransduced
TAL1-long
TAL1-short
49%
20%
28%
58%
18%
21%
43%
R3
23%
29%
Ly6G
R2
R1
Ly6C
C
E
D
3%
3%
37%
43%
12%
S2
4%
3%
55%
26%
9%
3%
1%
57%
12%
24%
S1
S3
S0
S4/5
CD71
Ter119
Untransduced
TAL1-long
TAL1-short
F
G
3%
0.2%
88%
8%
1%
0.2%
98%
0.3%
2%
0%
98%
0.8%
Thy1.2
CD19
I
H
53%
62%
63%
63%
Count
CD11b

## Slide 10
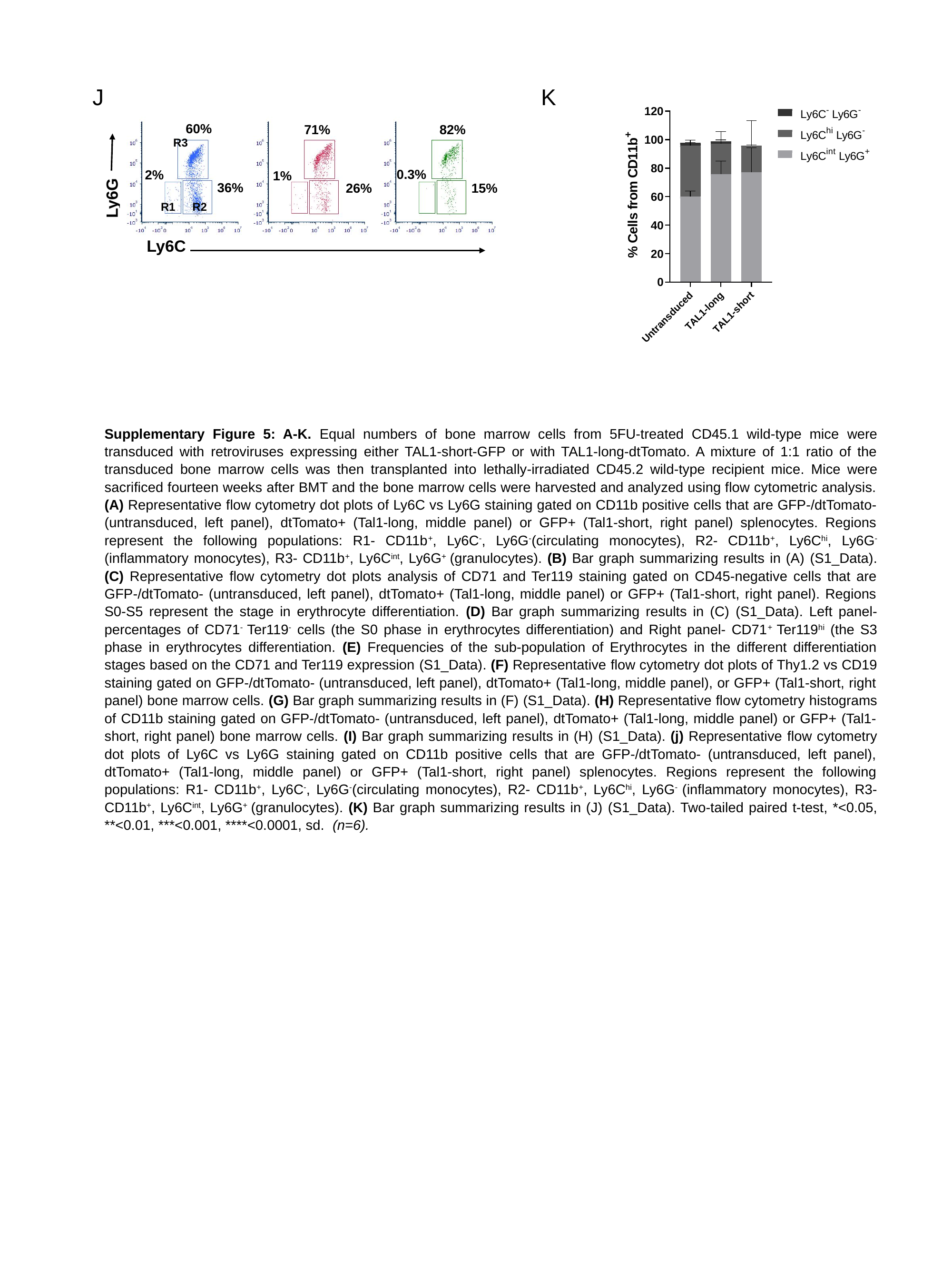

J
K
60%
2%
36%
71%
1%
26%
82%
0.3%
15%
R3
Ly6G
R2
R1
Ly6C
Supplementary Figure 5: A-K. Equal numbers of bone marrow cells from 5FU-treated CD45.1 wild-type mice were transduced with retroviruses expressing either TAL1-short-GFP or with TAL1-long-dtTomato. A mixture of 1:1 ratio of the transduced bone marrow cells was then transplanted into lethally-irradiated CD45.2 wild-type recipient mice. Mice were sacrificed fourteen weeks after BMT and the bone marrow cells were harvested and analyzed using flow cytometric analysis. (A) Representative flow cytometry dot plots of Ly6C vs Ly6G staining gated on CD11b positive cells that are GFP-/dtTomato- (untransduced, left panel), dtTomato+ (Tal1-long, middle panel) or GFP+ (Tal1-short, right panel) splenocytes. Regions represent the following populations: R1- CD11b+, Ly6C-, Ly6G-(circulating monocytes), R2- CD11b+, Ly6Chi, Ly6G- (inflammatory monocytes), R3- CD11b+, Ly6Cint, Ly6G+ (granulocytes). (B) Bar graph summarizing results in (A) (S1_Data). (C) Representative flow cytometry dot plots analysis of CD71 and Ter119 staining gated on CD45-negative cells that are GFP-/dtTomato- (untransduced, left panel), dtTomato+ (Tal1-long, middle panel) or GFP+ (Tal1-short, right panel). Regions S0-S5 represent the stage in erythrocyte differentiation. (D) Bar graph summarizing results in (C) (S1_Data). Left panel- percentages of CD71- Ter119- cells (the S0 phase in erythrocytes differentiation) and Right panel- CD71+ Ter119hi (the S3 phase in erythrocytes differentiation. (E) Frequencies of the sub-population of Erythrocytes in the different differentiation stages based on the CD71 and Ter119 expression (S1_Data). (F) Representative flow cytometry dot plots of Thy1.2 vs CD19 staining gated on GFP-/dtTomato- (untransduced, left panel), dtTomato+ (Tal1-long, middle panel), or GFP+ (Tal1-short, right panel) bone marrow cells. (G) Bar graph summarizing results in (F) (S1_Data). (H) Representative flow cytometry histograms of CD11b staining gated on GFP-/dtTomato- (untransduced, left panel), dtTomato+ (Tal1-long, middle panel) or GFP+ (Tal1-short, right panel) bone marrow cells. (I) Bar graph summarizing results in (H) (S1_Data). (j) Representative flow cytometry dot plots of Ly6C vs Ly6G staining gated on CD11b positive cells that are GFP-/dtTomato- (untransduced, left panel), dtTomato+ (Tal1-long, middle panel) or GFP+ (Tal1-short, right panel) splenocytes. Regions represent the following populations: R1- CD11b+, Ly6C-, Ly6G-(circulating monocytes), R2- CD11b+, Ly6Chi, Ly6G- (inflammatory monocytes), R3- CD11b+, Ly6Cint, Ly6G+ (granulocytes). (K) Bar graph summarizing results in (J) (S1_Data). Two-tailed paired t-test, *<0.05, **<0.01, ***<0.001, ****<0.0001, sd. (n=6).

## Slide 11
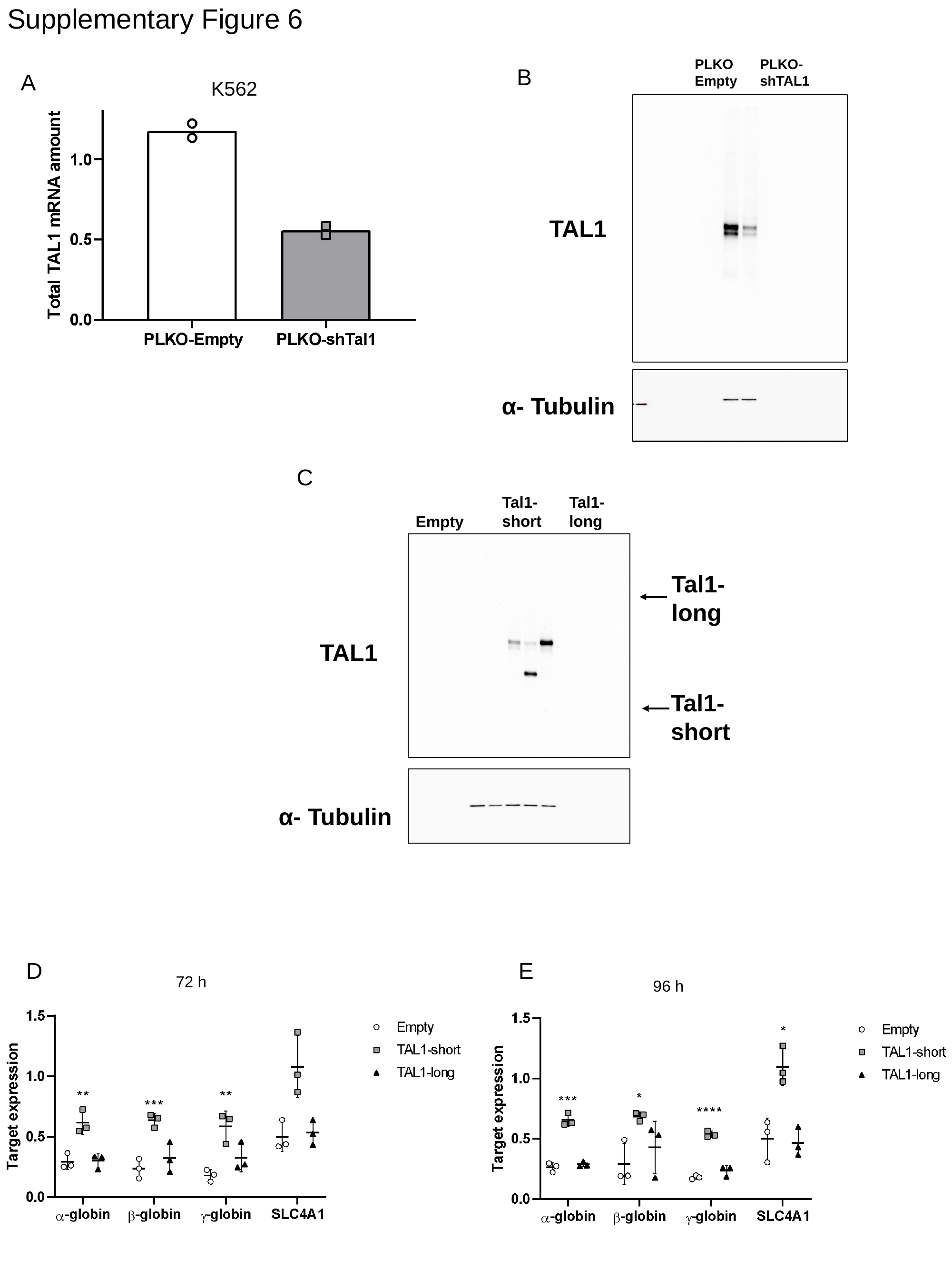

Supplementary Figure 6
PLKO
Empty
PLKO-shTAL1
B
A
K562
TAL1
α- Tubulin
C
Tal1-short
Tal1-long
Empty
Tal1-long
TAL1
Tal1- short
α- Tubulin
D
E
72 h
96 h

## Slide 12
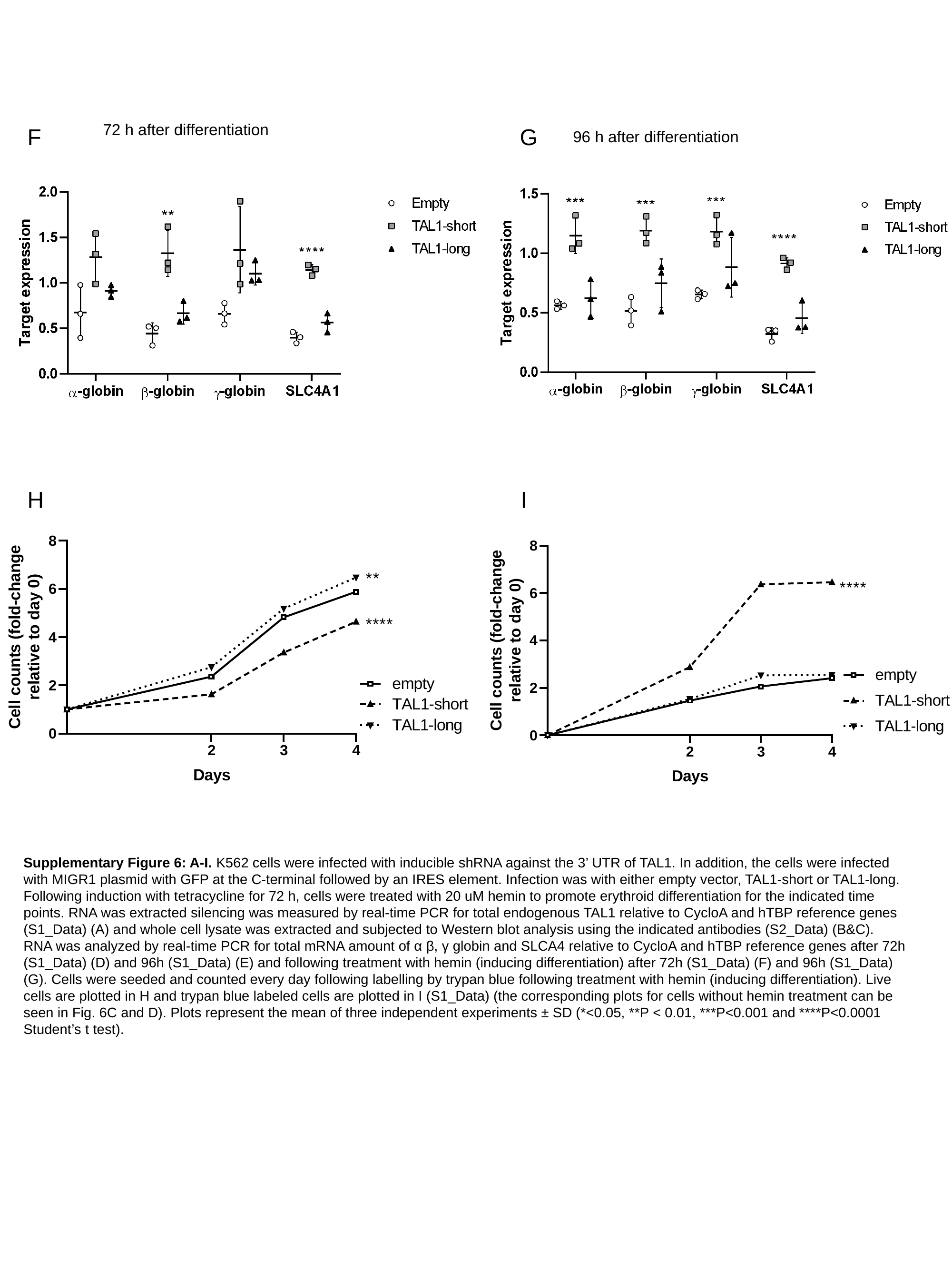

72 h after differentiation
F
G
96 h after differentiation
I
H
Supplementary Figure 6: A-I. K562 cells were infected with inducible shRNA against the 3’ UTR of TAL1. In addition, the cells were infected with MIGR1 plasmid with GFP at the C-terminal followed by an IRES element. Infection was with either empty vector, TAL1-short or TAL1-long. Following induction with tetracycline for 72 h, cells were treated with 20 uM hemin to promote erythroid differentiation for the indicated time points. RNA was extracted silencing was measured by real-time PCR for total endogenous TAL1 relative to CycloA and hTBP reference genes (S1_Data) (A) and whole cell lysate was extracted and subjected to Western blot analysis using the indicated antibodies (S2_Data) (B&C). RNA was analyzed by real-time PCR for total mRNA amount of α β, γ globin and SLCA4 relative to CycloA and hTBP reference genes after 72h (S1_Data) (D) and 96h (S1_Data) (E) and following treatment with hemin (inducing differentiation) after 72h (S1_Data) (F) and 96h (S1_Data) (G). Cells were seeded and counted every day following labelling by trypan blue following treatment with hemin (inducing differentiation). Live cells are plotted in H and trypan blue labeled cells are plotted in I (S1_Data) (the corresponding plots for cells without hemin treatment can be seen in Fig. 6C and D). Plots represent the mean of three independent experiments ± SD (*<0.05, **P < 0.01, ***P<0.001 and ****P<0.0001 Student’s t test).
