# Supplementary material for: Isoforms of the TAL1 transcription factor have different roles in hematopoiesis and cell growth
Source: PLoS Biol. 2023 Jun 28;21(6):e3002175. doi: 10.1371/journal.pbio.3002175 (PMC10335695; doi:10.1371/journal.pbio.3002175)
Supplement: S6 Fig — (A-I) K562 cells were infected with inducible shRNA against the 3′ UTR of TAL1. In addition, the cells were infected with MIGR1 plasmid with GFP at the C-terminal followed by an IRES element. Infection was with either empty vector, TAL1-short, or TAL1-long. Following induction with tetracycline for 72 h, cells were treated with 20 uM hemin to promote erythroid differentiation for the indicated time points. RNA was extracted silencing was measured by real-time PCR for total endogenous TAL1 relative to CycloA and hTBP reference genes (S1 Data) (A) and whole cell lysate was extracted and subjected to western blot analysis using the indicated antibodies (S1 Raw Images) (B and C). RNA was analyzed by real-time PCR for total mRNA amount of α-, β-, and γ-globin and SLCA4 relative to CycloA and hTBP reference genes after 72 h (S1 Data) (D) and 96 h (S1 Data) (E) and following treatment with hemin (inducing differentiation) after 72 h (S1 Data) (F) and 96 h (S1 Data) (G). Cells were seeded and counted every day following labelling by trypan blue following treatment with hemin (inducing differentiation). Live cells are plotted in H and trypan blue-labeled cells are plotted in I (S1 Data) (the corresponding plots for cells without hemin treatment can be seen in Fig 6C and 6D). Plots represent the mean of 3 independent experiments ± SD (*<0.05, **P < 0.01, ***P < 0.001, and ****P < 0.0001, Student t test). (PPTX) [file pbio.3002175.s006.pptx]

## Slide 1
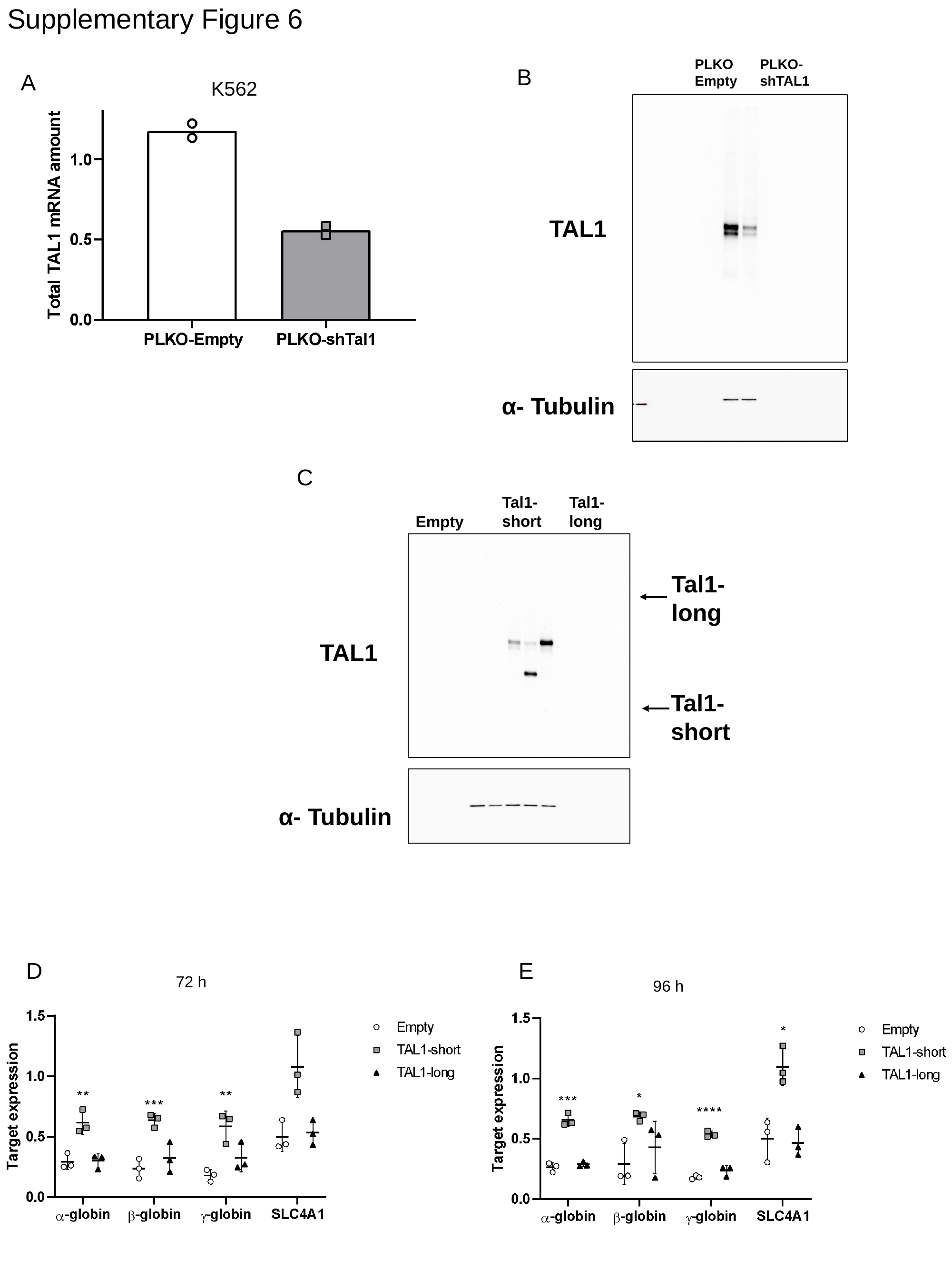

Supplementary Figure 6
PLKO
Empty
PLKO-shTAL1
B
A
K562
TAL1
α- Tubulin
C
Tal1-short
Tal1-long
Empty
Tal1-long
TAL1
Tal1- short
α- Tubulin
D
E
72 h
96 h

## Slide 2
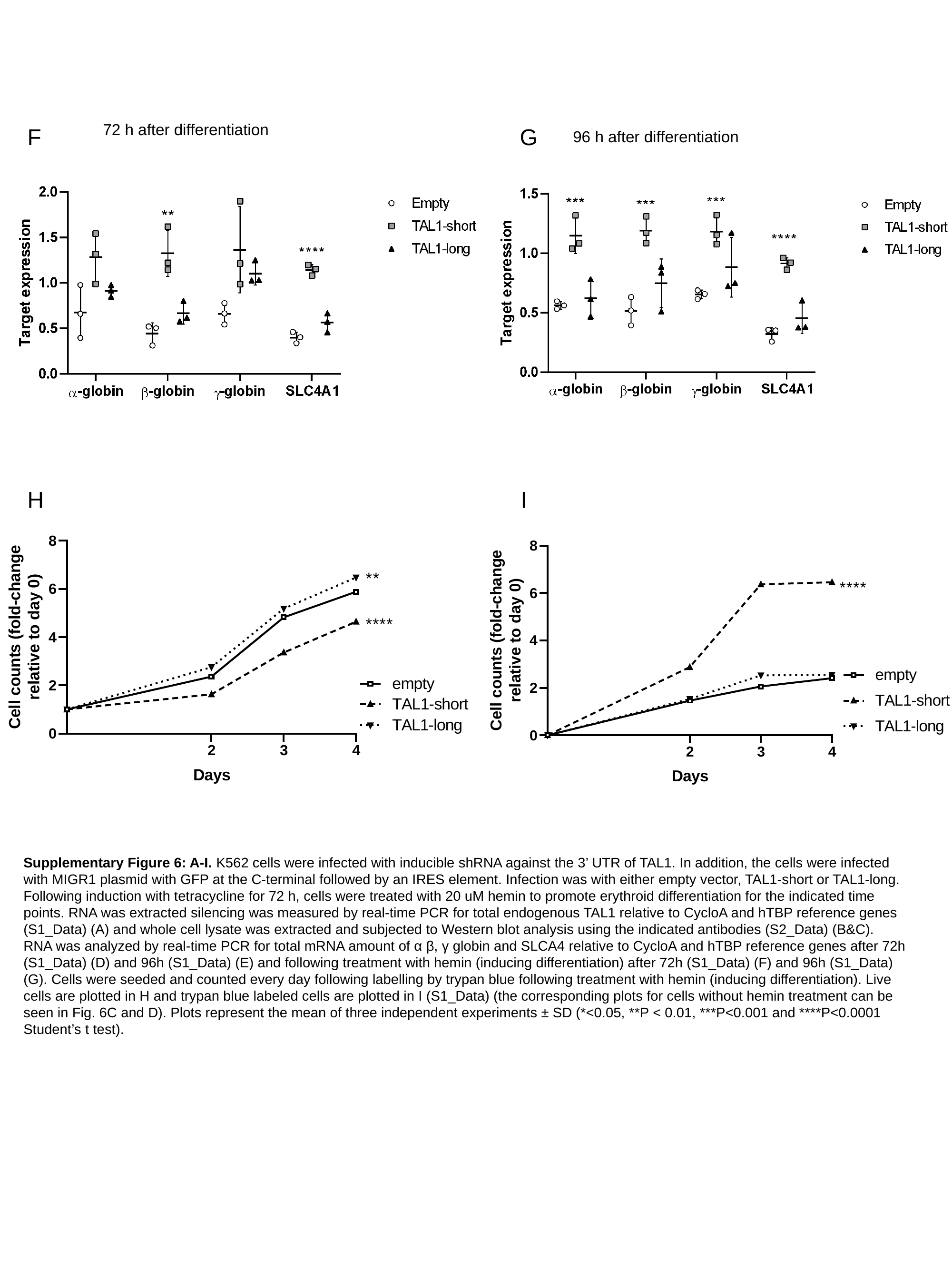

72 h after differentiation
F
G
96 h after differentiation
I
H
Supplementary Figure 6: A-I. K562 cells were infected with inducible shRNA against the 3’ UTR of TAL1. In addition, the cells were infected with MIGR1 plasmid with GFP at the C-terminal followed by an IRES element. Infection was with either empty vector, TAL1-short or TAL1-long. Following induction with tetracycline for 72 h, cells were treated with 20 uM hemin to promote erythroid differentiation for the indicated time points. RNA was extracted silencing was measured by real-time PCR for total endogenous TAL1 relative to CycloA and hTBP reference genes (S1_Data) (A) and whole cell lysate was extracted and subjected to Western blot analysis using the indicated antibodies (S2_Data) (B&C). RNA was analyzed by real-time PCR for total mRNA amount of α β, γ globin and SLCA4 relative to CycloA and hTBP reference genes after 72h (S1_Data) (D) and 96h (S1_Data) (E) and following treatment with hemin (inducing differentiation) after 72h (S1_Data) (F) and 96h (S1_Data) (G). Cells were seeded and counted every day following labelling by trypan blue following treatment with hemin (inducing differentiation). Live cells are plotted in H and trypan blue labeled cells are plotted in I (S1_Data) (the corresponding plots for cells without hemin treatment can be seen in Fig. 6C and D). Plots represent the mean of three independent experiments ± SD (*<0.05, **P < 0.01, ***P<0.001 and ****P<0.0001 Student’s t test).
