# Supplementary material for: Isoforms of the TAL1 transcription factor have different roles in hematopoiesis and cell growth
Source: PLoS Biol. 2023 Jun 28;21(6):e3002175. doi: 10.1371/journal.pbio.3002175 (PMC10335695; doi:10.1371/journal.pbio.3002175)

Raw immunoblots

Related to Fig 1C

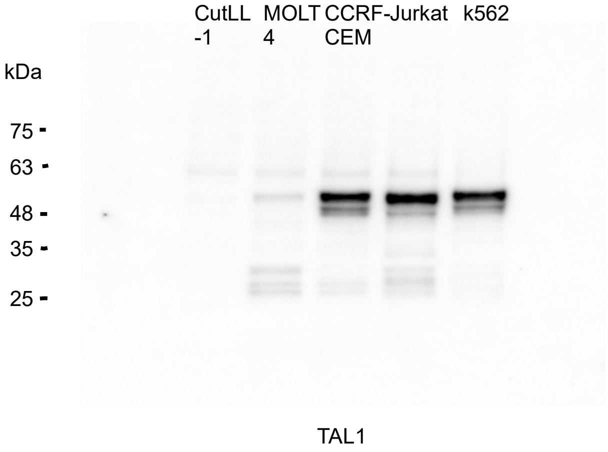

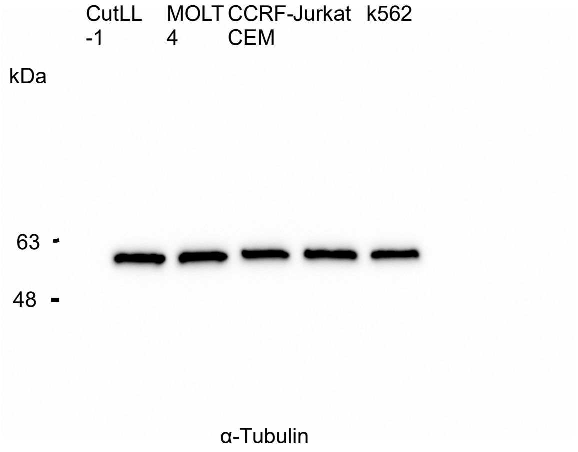

Related to Fig 4A

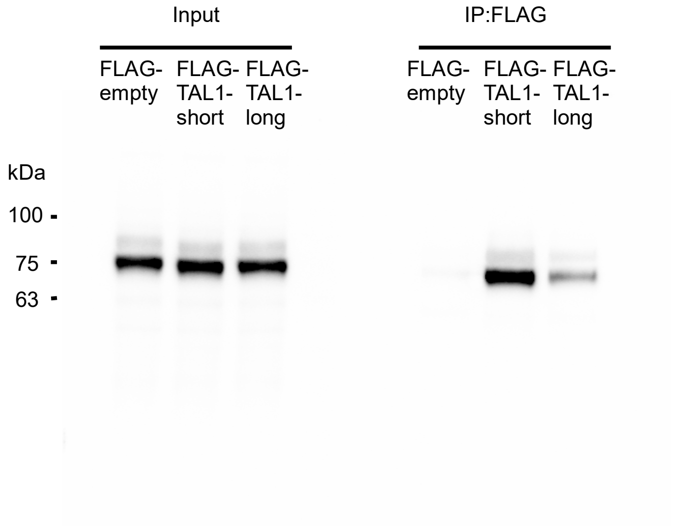

TCF-3

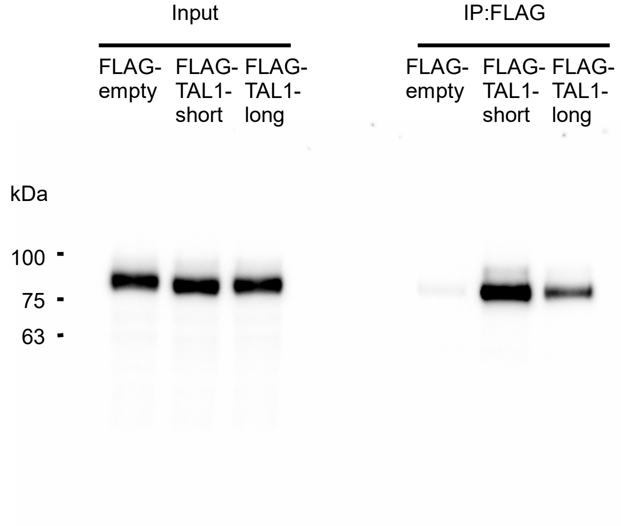

TCF-12

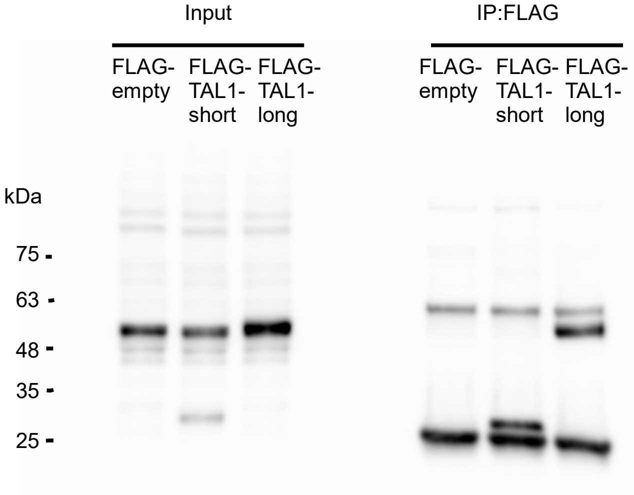

TAL1

Related to Supplementary Fig 2C

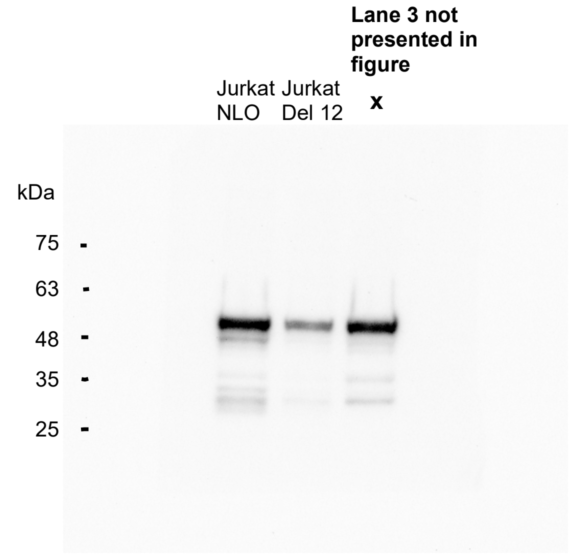

TAL1

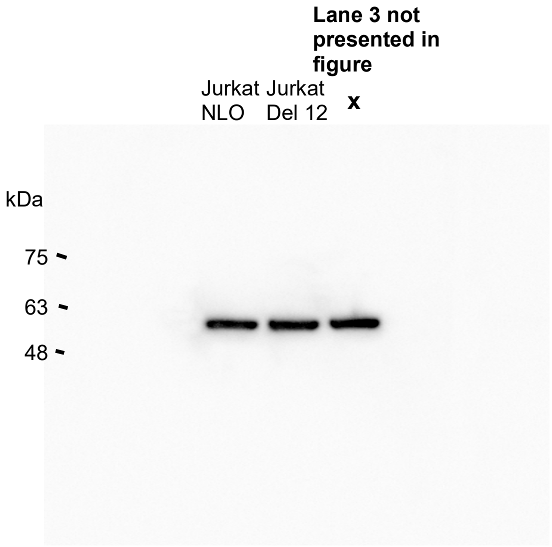

α-Tubulin

Related to Supplementary Fig 2F

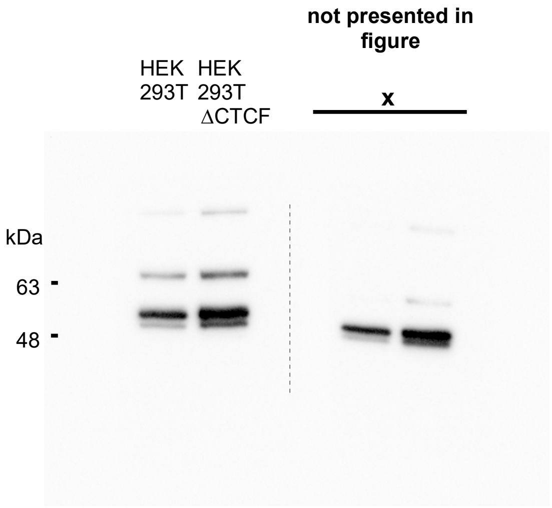

TAL1

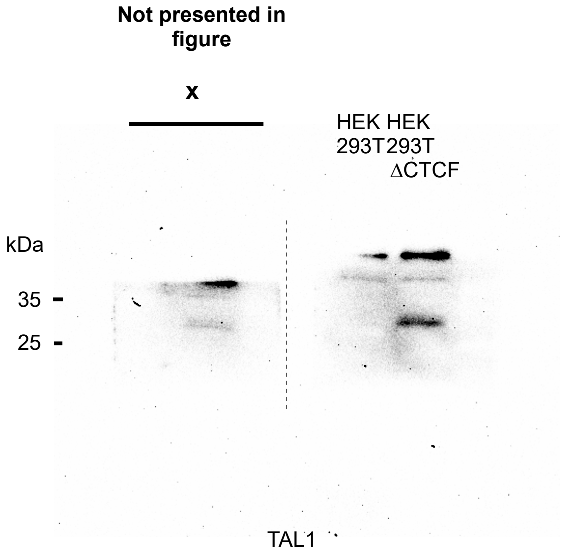

TAL1

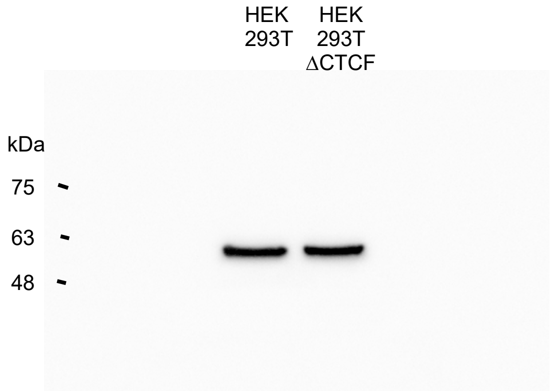

α-Tubulin

Related to Supplementary Fig 2H

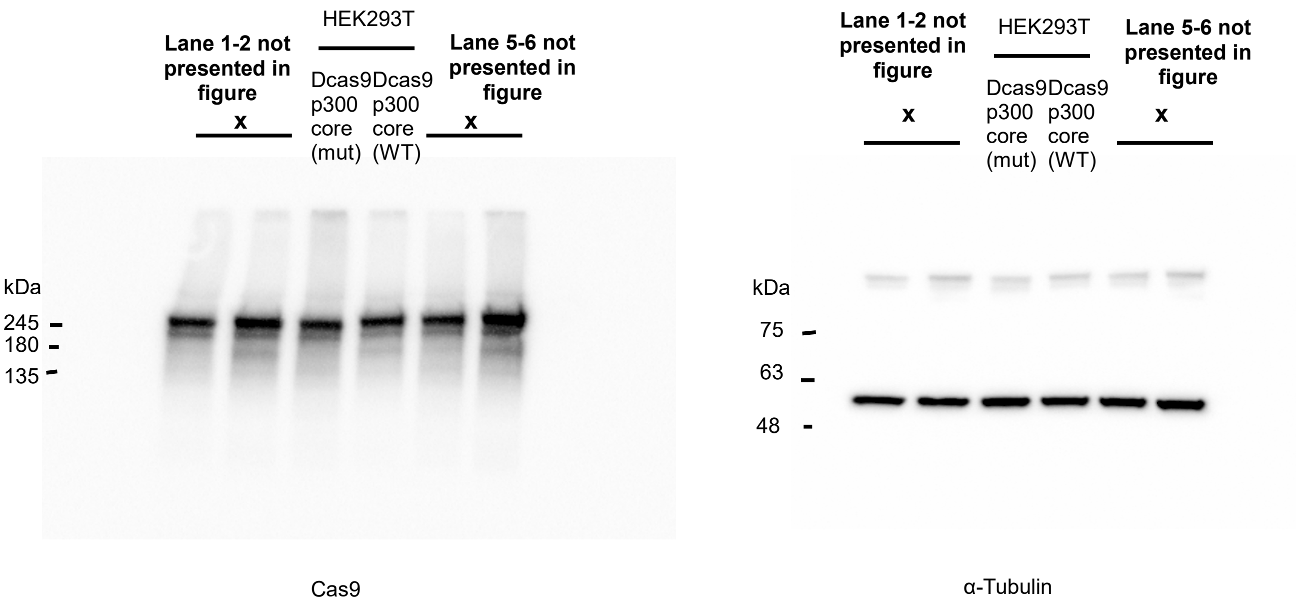

Related to Supplemental Fig 3B

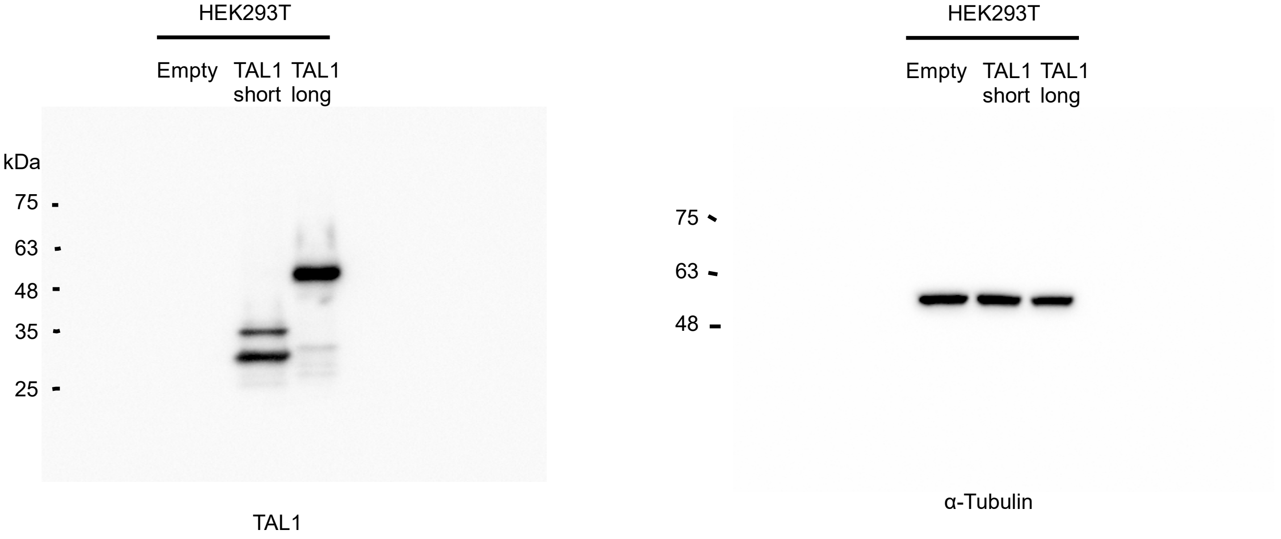

Related to Supplementary Fig 4A

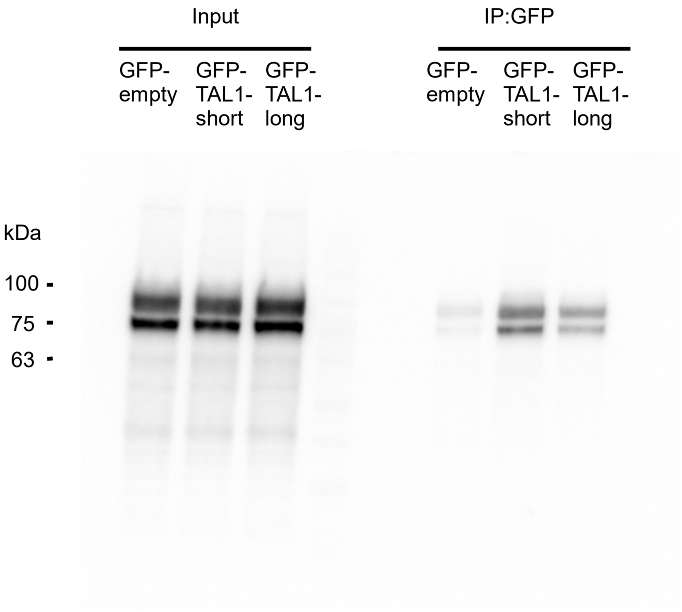

TCF-3

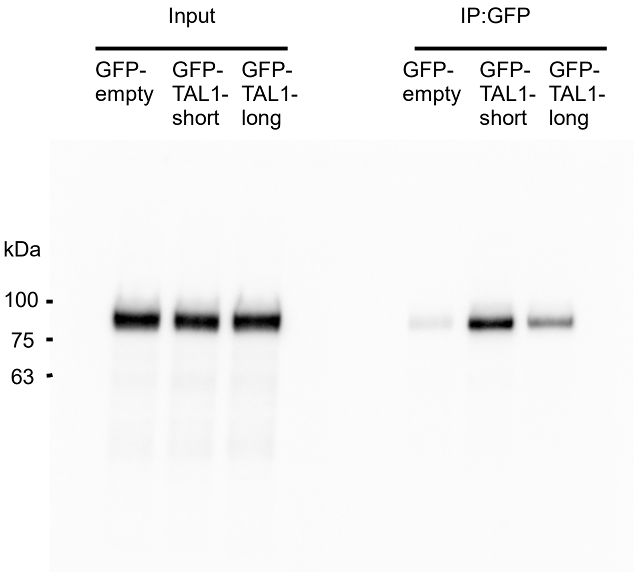

TCF-12

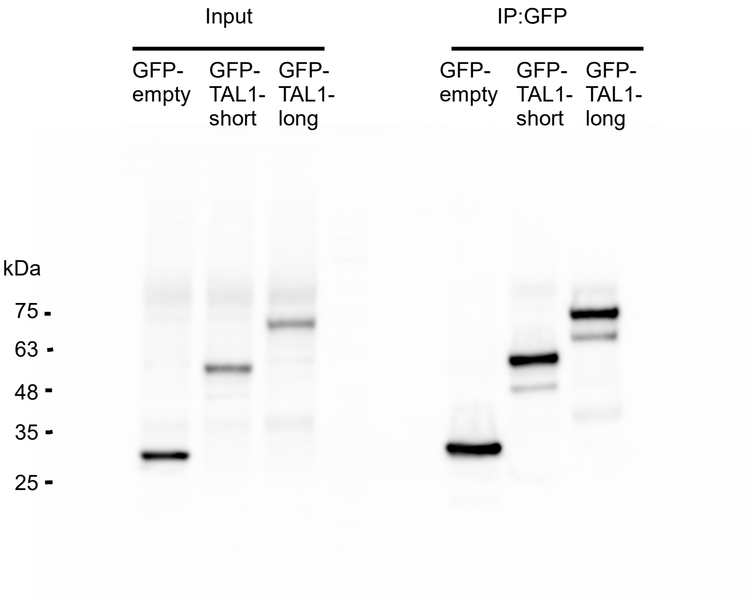

GFP

Related to Supplementary Fig 4B

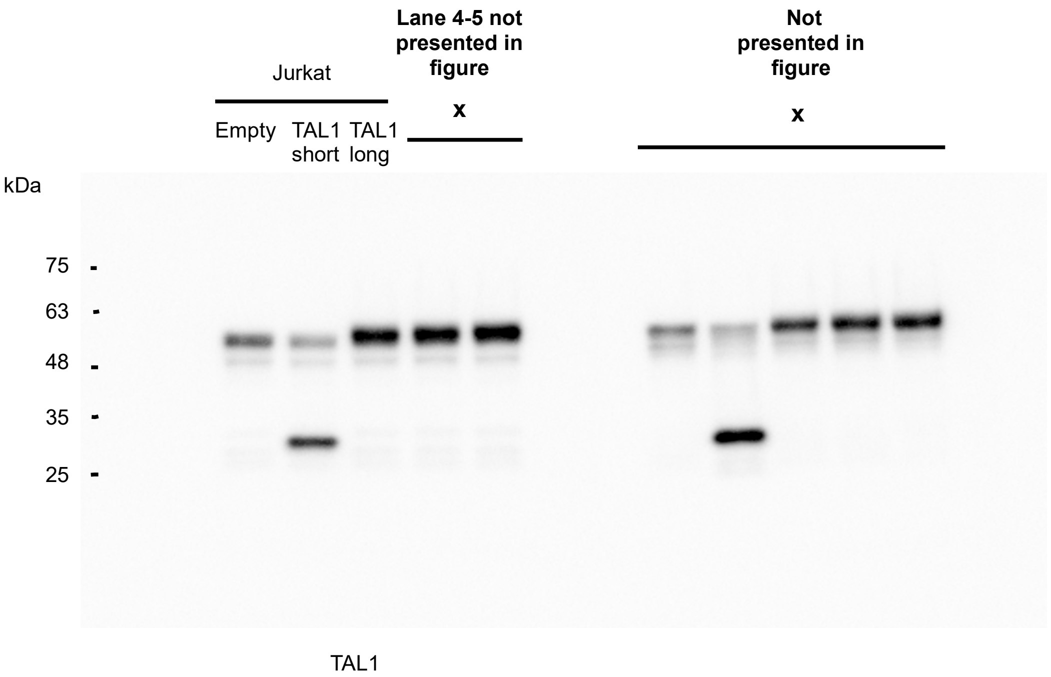

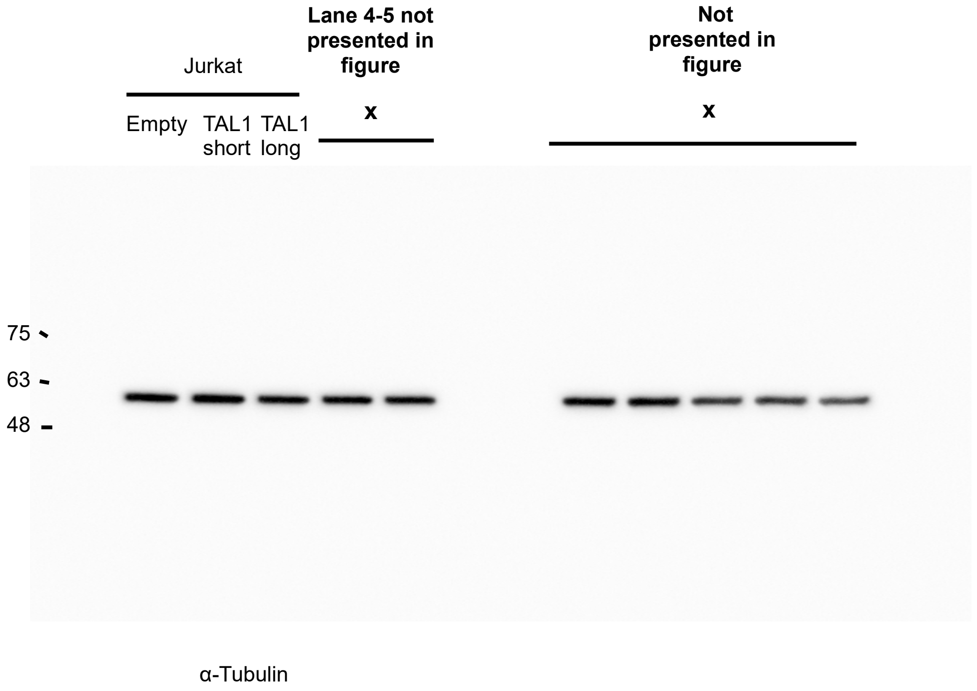

Related to Supplementary Fig 6B

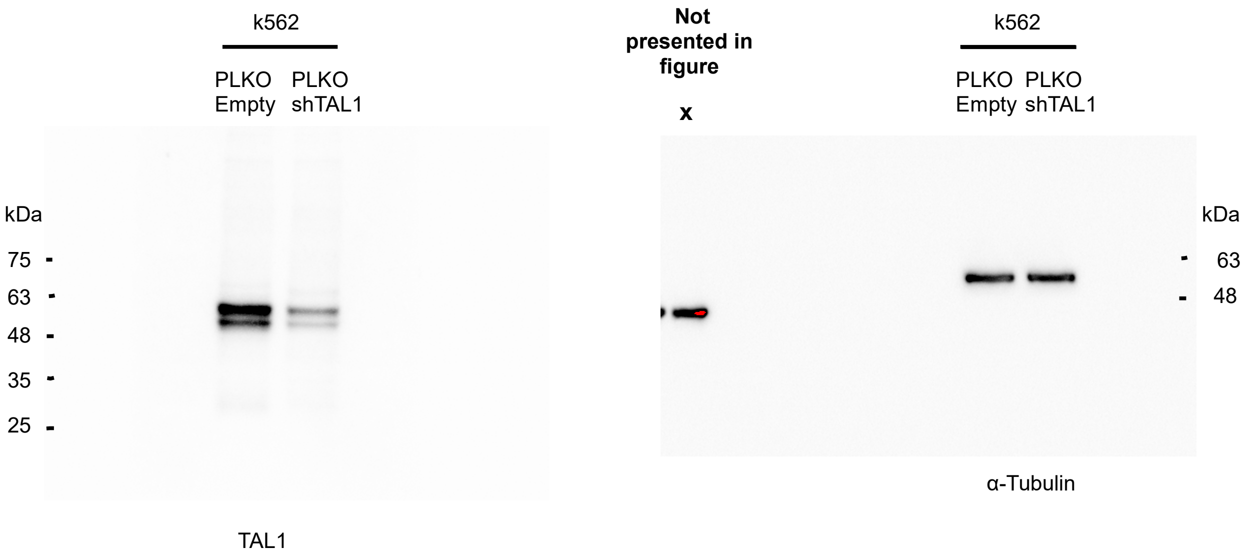

Related to Supplementary Fig 6C

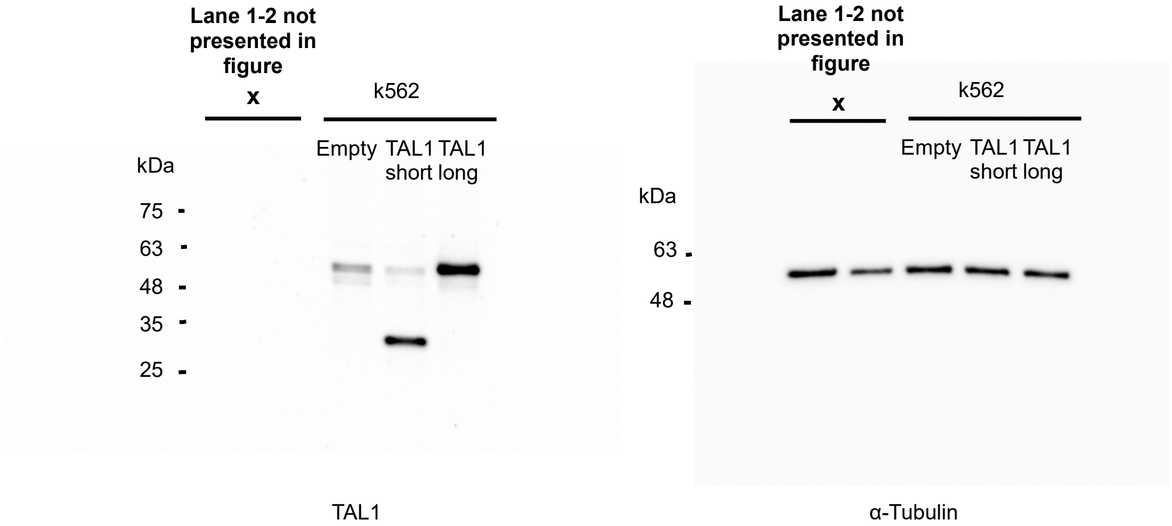

Supplement: S1 Raw Images — (PDF) [file pbio.3002175.s007.pdf]
