# Supplementary material for: Isoforms of the TAL1 transcription factor have different roles in hematopoiesis and cell growth
Source: PLoS Biol. 2023 Jun 28;21(6):e3002175. doi: 10.1371/journal.pbio.3002175 (PMC10335695; doi:10.1371/journal.pbio.3002175)
Supplement: S1 Table — (PDF) [file pbio.3002175.s008.pdf]

Supplementary Table S1 Primers used in this study		
**Primer name**	**Description**	**Sequence**
F_CyclophA	Endogenouse control for qPCR	GTCAACCCCACCGTGTTCTT
R_CyclophA		CTGCTGTCTTTGGGACCTTGT
F_hTBP	Endogenouse control for qPCR	CGCCAGCTTCGGAGAGTTC
R_hTBP		ACAACCAAGATTCACTGTGGATACA
F_TAL1_total	TAL1-total mRNA	GGAGCCGGATGCCTTCCCTAT
R_TAL1 total		GCTCCCGGCTGTTGGTGAAG
F_TAL1_3'TUR	TAL1 3' UTR	CTAGTGGCTTGTCCTCACC
R_TAL1_3'TUR		TCACAGGATCTTCCAAACGT
F_TAL1_ΔEx3	TAL ΔExon 3 mRNA	CTAAATATGCCCCAGCGGGT
R_TAL1_ΔEx3		CATCAGTAATCTCCATCTC
F_TAL1_TSS1-4	TAL1-long mRNA	AAAAAGGGGGAAAGCAAAGA
R_TAL1_TSS1-4		CCTCTAGCTGGGGGTCACT
F_TAL1_Exo	Exogenous+Endogenous TAL1 with MIGR1 infection	TGGAGATTACTGATGGTCCCC
R_TAL1_Exo		TCATTCTTGCTGAGCTTCTTGT
F_TSS1	TAL1 TSS1	TCAGGACCGGGCCTCAAAATG
R_TSS1		GGTGTGGTCCTGGGCGATGTT
F_TSS2	TAL1 TSS2	CATGCGGTGGGATTGTGAGAATC
R_TSS2		TGAATAGGATCTCCACTCCGCC
F_TSS4	TAL1 TSS4	AGCGTAACTGCAGGCCTCT
R_TSS4		TGAATAGGATCTCCACTCCGCC
F_Luciferase	Luciferase mRNA	GGACTTGGACACCGGTAAGA
R_Luciferase		CTTGTCGATGAGAGCGTTTG
F_Renilla	Renilla mRNA	GGAATTATAATGCTTATCTACGTGC
R_Renilla		CTTGCGAAAAATGAAGACCTTTTAC
F_gRNA_-60_Enh_1	gRNA target sequences for -60 Enhancer	CACCGCGGAGTTTAGGGAACCAGA
R_gRNA_-60_Enh_1		AAACTCTGGTTCCCTAAACTCCGC
F_gRNA_-60_Enh_2		CACCCTGGTCGTGTTCGTCCTCGG
R_gRNA_-60_Enh_2		AAACCCGAGGACGAACACGACCAG
F_gRNA_-60_Enh_3		CACCGGGACTCGTAGTCTGCGGCT
R_gRNA_-60_Enh_3		AAACAGCCGCAGACTACGAGTCCC
F_gRNA_-60_Enh_4		CACCTTAGCGTGTCGCTGATCGTG
R_gRNA_-60_Enh_4		AAACCACGATCAGCGACACGCTAA
F_gRNA_Ex3_1		CACCGCGGCCCTTTAAGTCTCTCG

R_gRNA_Ex3_1	gRNA target sequences for TAL1 Exon 3	AAACCGAGAGACTTAAAGGGCCGC
F_gRNA_Ex3_2		CACCGCGCCCAGTTCGATGACTGG
R_gRNA_Ex3_2		AAACCCAGTCATCGAACTGGGCGC
F_gRNA_Ex3_3		CACCGAGGACTCAGCTGCACCATG
R_gRNA_Ex3_3		AAACCATGGTGCAGCTGAGTCCTC
F_gRNA_Ex3_4		CACCCGTCCCGTCCCTCTAGCTGG
R_gRNA_Ex3_4		AAACCCAGCTAGAGGGACGGGACG
F_-60_Enhancer_ChIP	TAL1 -60 Enhancer for ChIP PCR	GAGGTTAGCGTGTCGCTGAT
R_-60_Enhancer_ChIP		AGACACGTTCGGTGACAGTG
F_TAL1_Exon3_ChIP	TAL1 Exon 3 for ChIP PCR	AGGAGGACGTGGACACCTTC
R_TAL1_Exon3_ChIP		CCAGCTAGCGCTGAGTTTCC
F_ α_Globin	Erythroid Lineage markers	CTGGAGAGGATGTTCCTGTCCTTG
R_ α_Globin		CAGCTTAACGGTATTTGGAGGTCAT
F_ β_Globin		ACTTCAGGCTCCTGGGCAAC
R_ β_Globin		GGCCCTTCATAATATCCCCCAG
F_ γ_Globin		GGACAAGGCTACTATCACAA
R_ γ_Globin		CAGTGGTATCTGGAGGACAG
F_SLC4A1		TGACCGCATCTTGCTTCTGT
R_SLC4A1		GTCTTCACCCGCTTGACGTA
F_TRIB2	TAL1 known Target genes	TGGGGACATGCATTCCTTCG
R_TRIB2		GACCCGAGTCCTCTCTTCGT
F_NFKB1		CGCCGCTTAGGAGGGAGA
R_NFKB1		CCATTCTGAAGCCGGGTGG
F_NKX3-1		CCTCCCTGGTCTCCGTGTA
R_NKX3-1		TGTCACCTGAGCTGGCATTAC
F_MLL4	MLL4 (KMT2D) Lysine methyltransferase 2D	TGCTATCACAGAAGCCCATG
R_MLL4		GGATCAATGATATCTTCTGCAG
F_18SrRNA	Human 18S rRNA (Endogenous control for CPT)	GGCCCTGTAATTGGAATGAGTC
R_18SrRNA		CCAAGATCCAACTACGAGCTT
