# Supplementary material for: Isoforms of the TAL1 transcription factor have different roles in hematopoiesis and cell growth
Source: PLoS Biol. 2023 Jun 28;21(6):e3002175. doi: 10.1371/journal.pbio.3002175 (PMC10335695; doi:10.1371/journal.pbio.3002175)
Supplement: Attachment — Submitted filename: 132652_1_rebuttal_2631133_rrx9n7.pdf [file pbio.3002175.s010.pdf]

Full Revision

Review COMMONS

Manuscript number: RC- 2022-01791

Corresponding author(s): Maayan, Salton

1. General Statements

The reviewers' comments and suggestions were extremely helpful in improving the clarity and quality of our work. We appreciate their time and effort in providing constructive feedback that allowed us to further develop our findings.

2. Point-by-point description of the revisions

Rebuttal Letter

Reviewer 1:

We would like to thank you for taking the time to review our manuscript. Your thoughtful and insightful comments have greatly improved the quality of our work. We appreciate your thoroughness in evaluating our study and providing valuable feedback.

Your constructive criticism and suggestions have helped us identify areas that needed further clarification and improvement, and we are grateful for your efforts in guiding us towards a stronger manuscript.

Thank you again for your time and expertise in reviewing our work. We hope that you find our revisions satisfactory and look forward to hearing your thoughts on the revised manuscript.

Reviewer #1 (Evidence, reproducibility and clarity (Required)):

In this manuscript by Sharma and colleagues, the authors investigate the transcriptional regulation of the TAL1 isoforms - that derive from differential promoter usage and/or alternative splicing - and the contribution of TAL1 long and TAL1 short protein isoforms in normal haematopoietic development and disease.

The study suggests that TAL1 transcript isoforms are fine-tuned regulated. By using CRISPR/Cas9 techniques, the authors show that the enhancer -8 (MuTE) and enhancer -60 differentially regulate the TAL1 isoforms. Whether the remaining enhancers at the TAL1 locus (see Zhou Y et al, Blood 2013) also differentially regulate TAL1 transcription remains to be elucidated.

The authors found that TAL1 short isoform interacts strongly with T-cell specific transcription factors such as TCF3 and TCF12, as compared to TAL1 long isoform. TAL1 short shows an apoptotic transcription signature and it fails in rescuing cell growth as compared to TAL1 long in T-ALL. In addition, TAL1 short promotes erythropoiesis.

Lastly, the authors suggest that altering TAL1 long and TAL1 short protein isoforms ratio could have a potential therapeutic application in disease, but further studies are needed.

We would like to thank you for your time and effort in reviewing our manuscript. Your constructive feedback and insightful comments have been immensely valuable in improving the quality of our work. Your expertise in the field has undoubtedly contributed to the credibility and accuracy of this research. In addition, your dedication and attention to detail have been instrumental in shaping the final version of the manuscript.

I have a number of comments:
Figure 1
It was not mentioned that MOLT4 cells also have MuTE. Do Jurkat and MOLT4 share a similar profile in terms of TAL1 transcript isoforms?
It would have been very interesting to see whether the TAL1 transcript isoforms are similar in SIL-TAL1+ cells (e.g RPMI-8402). In these cells, TAL1 activation results from a deletion that fuses the 5' non-coding region of SIL with TAL1.

Thank you for your comment. We apologize for the confusion regarding the MOLT4 cells in our analysis. We have now updated the manuscript to explicitly mention the presence of MuTE in MOLT4 cells (Line 127). Additionally, we agree that it would be interesting to investigate whether the TAL1 transcript isoforms are similar in SIL-TAL1+ cells, such as RPMI-8402. To address this point, we have included the CCRF-CEM cell line that harbors the SIL-TAL1 recombination in our analysis. We have updated the manuscript with these new findings (Fig. 1C&D and S1A&B). Thank you for bringing this to our attention.

Figure 2
It is not very clear how the expression of the short isoform delta exon 3 is quantified. Detailed information and a schematic of the primer location could be helpful.

Thank you for your comment. We apologize for any confusion regarding the quantification of the expression of the short isoform (delta exon 3). The detailed information and schematic of the primer location can be found in Supplementary Figure 2B. We have included the location of each primer used in real-time PCR analysis for the quantification of all TAL1 isoforms. We hope this additional information will address your concerns.

The results on Figure 2 derive from complex Cas9/CRISPR experiments. A schematic representation showing the location of the following elements is missing: CTCF sites, CTCF gRNA target region, dCas9-p300 gRNA target region and -60 enhancer.

We agree that providing a schematic representation of the Cas9/CRISPR experiments would be helpful for better understanding the data in Figure 2. We have now included a detailed schematic of the location of the CTCF sites, CTCF gRNA target region, dCas9-p300 gRNA target region and -60 enhancer in Supplementary Figure 2E. We believe this new figure will provide a clearer overview of the experiments performed and will aid in the interpretation of the results.

Are the levels of dCas9-p300 WT and dCas9-p300 MUT comparable in transfected HEK 293 cells? Were those possibly measured by qPCR or Western Blot? Why the authors chose to use 293T cells for the CTCF del as the enhancer usage around the locus must be so different from haematopoietic cells.

Thank you for your question. We have added Western Blot analysis to compare the levels of dCas9-p300 WT and dCas9-p300 MUT in transfected HEK293T cells, as suggested. The results are presented in Supp. Fig. S2H.

Regarding the choice of HEK293T cells for the CTCF deletion experiment, we selected this cell line for its low expression of TAL1, which contributes to a high dynamic range when tethering p300 core to a closed chromatin region. We have added a clarification of our rationale for using HEK293T cells in the revised manuscript (Lines 177-8). Thank you for your valuable feedback.

Is CPT - camptothecin? A control gene that is sensitive to CPT treatment would ensure the inhibitor is working.

Thank you for your comment. Indeed, CPT stands for camptothecin, and this information is already included in the methods section. We have also added this information to the results section (Line 221) to make it clearer.

Regarding the suggestion to use a control gene sensitive to CPT treatment, we agree that this could be a useful addition to our experimental design. To address this, we have quantified the amount of TAL1 transcript to an endogenous control which is not transcribed by RNA Polymerase II (RNAPII) (18s rRNA). As a positive control, we compared Cyclo A, our endogenous control, to 18s rRNA and observed a reduction (Supp. Fig. S2K). This allows us to confidently conclude that the inhibitor is working as intended.

Thank you for bringing up this point, and we hope that our response addresses your concern.

In supplementary Figure 2D, the reduction in expression in Jurkat Del-12 is restricted to TSS2. There is no reduction in TAL1 TSS1 and TAL1 TSS4 (this is not clear from the result description section). As seen, these isoforms are upregulated and that could suggest a compensatory mechanism mediated by alternative promoter activation. The fact that Jurkat Del-12 express TAL1 from MSCV-TAL1 could also suggest that TSS1 and TSS4 are upregulated by TAL1 or indirectly, by other members of the TAL/LMO complex (see Sanda T et al, Cancer Cell 2012)
Certainly, we appreciate your feedback. Supplementary Figure 2D indeed shows that the MuTE enhancer has a differential effect on the promoters, and we have now included this in the text of the manuscript. Regarding the TAL1-long isoform, while MSCV-TAL1 in the Jurkat Del-12 cell line does give rise to this isoform, our results from Figure 3A did not find TAL1-long to have a differential effect on TAL1 promoters. It is important to note that the experiment conducted was an exogenous construct in HEK293T cells, which has its limitations. Thus, the speculation that TAL1-long drives the result in supplementary Figure 2D is possible, and we have added this to the text. Thank you for bringing up this important point (Lines 167-9).

Figure 3
A. Are the levels of TAL1 short cDNA and TAL1 long cDNA comparable in the co-transfection luciferase experiments? The overexpression of the isoforms does not reflect the endogenous expression levels in cell lines where one of the isoforms is more predominantly expressed (e.g Jurkat cells express low levels of TAL1 short).
Thank you for your comment. To address your concern, we have added real time (Supp. Fig. S3A) as well as Western blot in a new figure (Supp. Fig. S3B) to show that the levels of TAL1-short and TAL1-long cDNA are comparable in the co-transfection luciferase experiments. Additionally, we observed a very low amount of endogenous TAL1 isoforms in the cell line (Supp. Fig. S3A&B), which was below detection using these methods. This suggests that the effect of the endogenous TAL1 in this cell line is low. We appreciate your feedback, and we hope this additional information addresses your concern.

Figure 4
Are the levels of flag-TAL1 long and flag-TAL1 short comparable? The levels of expression could explain the low intensity signal for TAL1 long.

Thank you for your insightful comment. Indeed, the issue of isoform quantification is critical in understanding the functional differences between TAL1-short and TAL1-long. To address this concern, we performed careful quantification of the isoforms and made sure that the amount

was equal or slightly in favor of TAL1-long before conducting the experiments in this manuscript. We have also added a Western blot in Supp. Fig. S3A and real time in Supp. Fig. S3B showing the similar amount of the two isoforms. Furthermore, in Figure 4A, we provided the amount of each isoform in the input section, showing a higher amount of TAL1-long. This strengthens our result, which shows that TAL1-short binds stronger to TCF-3 and 12. Protein levels for ChIP-seq experiment (Fig. 4B-H) is now in Supp. Fig. S4B. We thank you for bringing up this important point, and we hope that our additional data and clarifications have addressed your concern

Is there any reason for not performing a depletion of endogenous TAL1 prior to the ChIP seq flag experiment?

Thank you for your comment. In our experience, infecting Jurkat cells with shRNA or an expressing vector systems can induce some cellular stress, and we did not want to add additional stress to the cells by depleting endogenous TAL1. Since we immunoprecipitated using a Flag-tagged protein, we did not see a need to deplete the endogenous TAL1 protein. However, in our RNA-seq experiment, depletion of endogenous TAL1 was critical, and we have added this additional step in this experiment.

Could the authors speculate about MAF motif enrichment in both isoforms and not in TAL1-total?

Thank you for bringing up this interesting point. It is worth noting that while all ChIP-seq experiments were performed in Jurkat cells, not all of them were conducted by us. In particular, ChIP-seq of TAL1 total was performed by Sanda et al., 2012, using an endogenous antibody against both isoforms, whereas we conducted ChIP-seq for TAL1-short and TAL1-long using a FLAG tag antibody in cells expressing each of the isoforms. Therefore, the different conditions of these experiments may have contributed to the observed MAF motif enrichment in both isoforms and not in TAL1-total. While we cannot provide a definitive explanation, we speculate that the overexpression of the isoforms or the presence of the FLAG tag may have facilitated the detection of the MAF motif. We have added this discussion to the manuscript to acknowledge and address this interesting observation (Lines: 307-8).

T. Sanda et al., Core transcriptional regulatory circuit controlled by the TAL1 complex in human T cell acute lymphoblastic leukemia. Cancer Cell 22, 209-221 (2012).

Do TAL1 long and TAL1 short recognise the same DNA motif?

This is indeed a very interesting question but a difficult one to answer since TAL1 does not bind to the DNA alone but in a complex. In this situation, the ChIP-seq de-novo binding results suggest motifs that could be recognized by TAL1 or any of its complex partners. Using previous data, TAL1's binding motif is CAGNTG (Hsu et al., 1994), while this motif was not identified in our analysis of the TAL1-total or FLAG-TAL1-long ChIP-seq results, we did, however, identify this sequence in FLAG-TAL1-short ChIP-seq results (p value=1e-93). We predict that this discrepancy is due to the complex nature of transcription factors binding and the fact that the ChIP-seq results were not all done in the same way. We have now added this to the discussion (Lines: 419-25).

H. L. Hsu et al., Preferred sequences for DNA recognition by the TAL1 helix-loop-helix proteins. Mol Cell Biol 14, 1256-1265 (1994).

Figure 6
In A and B, are the levels of flag-TAL1 long and flag-TAL1 short in transduced K562 comparable? In C and D, are the TAL1 levels reduced at the protein level?
Thank you for your question. To answer your question, we added Western Blot analysis to show the comparable levels of flag-TAL1-long and flag-TAL1-short in transduced K562 cells (Supp. Fig. S6C). In Figure 6C and D, we also added Western Blot analysis to show the reduction in TAL1 protein levels upon shRNA-mediated knockdown (Supp. Fig. S6B).

Minor points:

Figure 1
A. Include a scale bar

To address this, we included coordinates of the components of the gene marked in the figure.

C. Loading control such as GAPDH is missing in the Western Blot. Are CUTLL cells the same as CUTTL-1?

We added loading controls as requested now supplementary Fig. 1C, S2C, S3A, S4B, S6B&C. Yes, CUTLL is the same as CUTLL-1 we have now fixed this in the text (Line 120).
D. Adjust scale of the CHIP seq tracks in K562 cells in order to see the peak summit. Include genome build

Thank you for your comment. We have adjusted the scale of the ChIP-seq tracks in K562 cells as suggested to improve the visualization of the peak summit. However, one of the peaks still had a much higher signal and the summit is still missing from this particular peak. To address this, we have added a new figure in the supp. Fig. S1C materials where we adjusted the peak to show the summit. Please note that in this track, the chromatin structure at the enhancers is missing, and therefore, we did not include it in the main figure. Thank you for bringing this to our attention.

We have added a genome build hg19 to the figure legend.

In supplementary Figure 1B, the symbol scheme is not clear
Thank you for this note, we have replaced the figure and added text to make it clearer.

Figure 2
A & C. Remove 'amount' from the Y axis. Is the total mRNA amount calculated as % of the reference genes? It could be specified on the y axis or figure legend.

We have removed the word "amount" from the Y axis as requested. Total mRNA amount is normalized relative to the reference genes (ΔΔCq) by Bio-Rad's CFX Maestro software (version 2.3) according to the formula:

$$\text{Normalized Expression}_{\text{sample (GOI)}} = \frac{RQ_{\text{sample (GOI)}}}{\left(RQ_{\text{sample (Ref 1)}} \times RQ_{\text{sample (Ref 2)}} \times \ldots \times RQ_{\text{sample (Ref n)}}\right)^{\frac{1}{n}}}$$

where:

- RQ = Relative Quantity of a sample
- Ref = Reference target in a run that includes one or more reference targets in each sample

- GOI = Gene of interest (one target)

In supplementary Figure 2C, a loading control is missing.
We have added alpha-tubulin to this figure.

Figures 4, 5 and 6
Size of the figures should be increased.
We have increased the figure size as suggested.

Reviewer #1 (Significance (Required)):
The study from Sharma and colleagues is novel and it extends the knowledge on TAL1 regulation and the role of TAL1 in development and disease. Although the study suggests that there is a correlation between enhancers, chromatin mark deposition at exons and regulation of alternative splicing, the mechanistic link is not fully elucidated.

To further elucidate the mechanistic link between the MuTE enhancer, broad H3K4me3 modification spanning 7.5 Kbp from TAL1 promoter 1 to promoter 5 (as shown in Fig. 1D), and alternative splicing, we conducted experiments where we manipulated KMT2B, a component of the SET1/COMPASS complexes responsible for methylating H3K4. Our findings indicate that silencing KMT2B in Jurkat cells led to a significant 30% increase in TAL1-ΔEx3 (Fig. 2H and Supp. Fig. S2I&J). These results contribute to a more comprehensive understanding of the molecular mechanisms underlying TAL1 alternative splicing regulation.

The findings on TAL1 short protein are interesting but the data on TAL1 long lacks some refinement so then robust conclusions can be drawn.
The experimental data lacks a few controls. The text is clear and prior studies could be better referenced.

We have made an effort to better reference out manuscript.

As TAL1 is a very crucial transcription factor oncogene in T-ALL, the study is important as it addresses a very relevant question in the field that is the regulation of the transcription of TAL1 and the functional relevance of both TAL1 short and TAL1 long isoforms.

Reviewer 2:

Reviewer #2 (Evidence, reproducibility and clarity (Required)):

Summary:
Sharma et al. thoroughly characterized the regulation of TAL1 by mapping the use of its five promoters and enhancers, which together transcribe five transcripts, coding for two protein isoforms. For that purpose the authors used few cell lines: Jurkat as a T-ALL cell line, chronic myeloid leukemia (CML) cell line K562 and HEK293T with low TAL1 expression, as well as CutLL and MOLT4.
They profiled the chromatin marks H3K27ac and H3K4me3 at the TAL1 locus, and show that when a the -8 enhancer is compromised tha chromatin marks change, and not only the expression level of TAL1 is reduced, the level of exon 3 skipping is increased. When the -60 enhancr was activated, TAL1 expression increased, and exon 3 skipping was reduced. Those findings indicate that in tal1, transcription and alternative splicing are co-regulated, independent

of RNAPII.
The authors also show that as an autoregulator, TAL1-short has a preference to TSS1-3 of TAL1, which is not shared by TAL1-long, and that each of the 5' UTR affect Tal1 expression differently. TAL1-short binds E-proteins more strongly than TAL1-long, binds many more sites than TAL1-long and stronger, and each isoform has unique set of targets.
Finally, the authors set to identify the different functions of the TAL1 isoforms, and showed that Tal1-short slows cell growth and leads to TAL1-short but not TAL1-long leads to exhaustion of hematopoietic stem cells and promotes differentiation into erythroids.
This paper used for the first time TAL1 isoform specific ChIP-seq, which enable accurate definition of isoform-specific targets in Jurkat cells. They demonstrated an interaction between choice of TSS and alternative splicing, and isoform specific functions. Given the clinical importance of TAL1 and the meticulous work performed to characterize its isoform specific regulation and function, I find this manuscript of interest, and only have minor suggestions to improve readability.

Thank you for taking the time to carefully review our manuscript on the regulation and function of TAL1 isoforms. We appreciate your positive feedback on our comprehensive characterization of TAL1 regulation using chromatin profiling and isoform-specific ChIP-seq. We are glad that you found our findings on the co-regulation of transcription and alternative splicing, as well as the isoform-specific functions of TAL1, to be of interest.

We also appreciate your suggestions to improve the readability of the manuscript and have made the necessary revisions accordingly. Your feedback has been invaluable in strengthening the quality of our work, and we are grateful for your contribution to the scientific community.

Minor comments:
Add explicitly the motivation for choosing the cell line in each part.

We have added motivation (Lines: 157-8, 177-8, 192-194, 235-6 text that was on the previous version: 192-194, 379-80).
Figure 1 - Consider marking the promoter numbers and the enhancers names in the same names as in text (-8,-60 etc.), to make it easier for the readers to understand which enhancers is being discussed.

This in a very important point. We have added the numbering to Figure 1D and Supp. Fig. S2A, B & E.
P5, P18 - ProtParam is only a prediction tool, and does not supply an experimental measurement, as may be assumed from text. Please rephrase accordingly.

The words “prediction tool” were added in the indicated paragraphs (Lines 115 and 427).
Figure 2B/D - y axis label unclear, not explained in text. In accordance, unclear if the change is in the amount of RNA, or the ratio between the long and short variants.

Thank you for this comment. We greatly appreciate your feedback and suggestions. To make our calculations, which are the norm in the splicing field, clearer, we have now added text to Figure 4 and provided more detailed explanations in lines 670-73. We hope that these modifications will improve the clarity and comprehensiveness of our manuscript.

Consider removing the bars and increasing the dots, to make the graphs cleaner.

We removed the bars throughout the manuscript for a cleaner look.
P8 - The term '5C' may require more explanation, depending on target audience.

We have added text to explain the technique (Lines 179-81).

Figure 3 - the trend is that TAL1-short promotes transcription from all five TSSs. However, only in TSS1-3 is the difference significant, but the difference between the long and short forms is not significant. It is unclear if "The mean of three independent experiments done with three replicates" means overall there are three replicates per condition or nine. Please rephrase to clarify.

Thank you for your comment. To clarify, we want to state that each biological experiment was done in three technical replicates, resulting in a total of nine replicates for each condition. We apologize for any confusion and have now rephrased to: The mean was calculated from three independent biological experiments, each performed with three technical replicates (Lines: 696 and 699).

Fig 4 A - it seems that many of the sites bound by Tal1 total are not bind by either Tal1-short or Tal1-long. Indeed very little overlap between Tal1-short and Tal-1-total is seen in Fig 4I as well. It seems Tal1-long has very few peaks. Consider adding a discussion of possible reasons.

We agree that these findings are noteworthy and warrant further discussion. We added text to the discussion section to explore potential reasons for these observations (Lines 416-25).

Fig 4c - it is hard to distinguish the different lines. Consider a more clear visualization. Also, some text is in a font size too small to read.

We have changed the format of the figure and took out the input data from the main figure to help the visualization. The input data appear in the Supp. Fig. S4C.

Fig 4 D-H - will be useful to see the numbers, not just the % divided by %.

A table with the specific numbers can be found in Supp Figure 4F-J.

Fig 4 legend - 'I&L' possibly means 'I-L'.
P14 - refer to where the results of the 'validation using real-time PCR' are shown.
P16 - symbol replaced by an empty rectangle 20 □M

Thank you for these valuable comments, we have fixed/added these in the manuscript.

Figure 6D - Y axis value seem strange (fold change relative to day 0 should be 1 at day 0). Consider different Y axis label for C and D to clarify.

Thank you for this comment, we have changed the y-axis to: Fold-change relative to day 1.

P18 - It is unclear which "two isoforms with posttranslational modifications which affected the migration rate of the protein (Fig. 1C)" were shown. Only two isoforms are mentioned throughout the paper.

We have added text to clarify we are referring to TAL1-short and long (Lines 409-10).

P18 - "Our ChIP-seq results suggest that the isoforms bind at the same location (Fig. 4B)." - in 4B it seems most of TAL1-short bound positions are not bound by TAL1 long. Please clarify. Worth mentioning that the Total TAL1 is taken from Jurkat cells but from a different experiment.

We have changed the statement and added the text referring to the experiments done

independently (Lines 422-3).

Reviewer #2 (Significance (Required)):
This paper used for the first time TAL1 isoform specific ChIP-seq, which enable accurate definition of isoform-specific targets in Jurkat cells. They demonstrated an interaction between choice of TSS and alternative splicing, and isoform specific functions. Given the clinical importance of TAL1 and the meticulous work performed to characterize its isoform specific regulation and function, I find this manuscript of interest, and only have minor suggestions to improve readability.
